# DE-PASS Best Evidence Statement (BESt): Determinants of self-report physical activity and sedentary behaviours in children in settings: A systematic review and meta-analyses

**DOI:** 10.1371/journal.pone.0309890

**Published:** 2024-11-25

**Authors:** Fiona C. M. Ling, Mohammed Khudair, Kwok Ng, Gavin D. Tempest, Ratko Peric, František Bartoš, Maximilian Maier, Mirko Brandes, Angela Carlin, Simone Ciaccioni, Cristina Cortis, Chiara Corvino, Andrea Di Credico, Patrik Drid, Francesca Gallè, Pascal Izzicupo, Henriette Jahre, Athanasios Kolovelonis, Atle Kongsvold, Evangelia Kouidi, Paul J. Mork, Federico Palumbo, Penny L. S. Rumbold, Petru Sandu, Mette Stavnsbo, Ioannis Syrmpas, Sofia Vilela, Catherine Woods, Kathrin Wunsch, Laura Capranica, Ciaran MacDonncha, Anna Marcuzzi

**Affiliations:** 1 Department of Sport, Exercise and Rehabilitation, Northumbria University, Newcastle Upon Tyne, United Kingdom; 2 Department of Education, University of Turku, Rauma, Finland; 3 Department of Physical Education and Sport Sciences, University of Limerick, Limerick, Ireland; 4 Institute of Innovation and Sports Science, Lithuanian Sport University, Kaunas, Lithuania; 5 Exercise Physiology Laboratory, OrthoSport Banja Luka, Banja Luka, Bosnia; 6 Department of Psychological Methods, University of Amsterdam, Amsterdam, Netherlands; 7 Department of Experimental Psychology, University College London, London, United Kingdom; 8 Department of Prevention and Evaluation, Leibniz Institute for Prevention Research and Epidemiology–BIPS, Bremen, Germany; 9 Centre for Exercise Medicine, Physical Activity and Health, Sport and Exercise Sciences Research Institute, Ulster University, Belfast, Ireland; 10 Department of Movement, Human and Health Sciences, University of Rome "Foro Italico", Rome, Italy; 11 Department of Human Sciences, Society and Health, University of Cassino and Lazio Meridionale, Cassino, Italy; 12 Department of Psychology, Faculty of Economics, Università Cattolica del Sacro Cuore, Milan, Italy; 13 Department of Medicine and Aging Sciences, University “G. d’Annunzio” of Chieti-Pescara, Pescara, Italy; 14 Faculty of Sport and Physical Education, University of Novi Sad, Novi Sad, Serbia; 15 Department of Movement Sciences and Wellbeing, University of Naples Parthenope, Naples, Italy; 16 Department of Rehabilitation Science and Health Technology, Oslo Metropolitan University, Oslo, Norway; 17 Department of Physical Education and Sport Sciences, University of Thessaly, Thessaly, Greece; 18 Department of Public Health and Nursing, Norwegian University of Science and Technology, Trondheim, Norway; 19 Laboratory of Sports Medicine, Department of Physical Education and Sports Science, Aristotle University of Thessaloniki, Thessaloniki, Greece; 20 Health Promotion and Evaluation, National Institute of Public Health in Romania, Bucharest, Romania; 21 Department of Sport Science and Physical Education, Faculty of Health and Sport Sciences, University of Agder, Kristiansand, Norway; 22 EPIUnit—Institute of Public Health, University of Porto, Porto, Portugal; 23 Institute of Sports and Sports Science, Karlsruhe Institute of Technology, Karlsruhe, Germany; University of the Witwatersrand Johannesburg, SOUTH AFRICA

## Abstract

Previous physical activity interventions for children (5-12yrs) have aimed to change determinants associated with self-report physical activity behaviour (PAB) and/or sedentary behaviour (SB), however, the associations between these determinants and PAB/SB in different settings are uncertain. The present study aimed to identify modifiable determinants targeted in previous PAB/SB interventions for children. Intervention effects on the determinants and their associations with self-report PAB/SB were assessed across settings. Search of relevant interventions from pre-defined databases was conducted up to July 2023. Randomized and non-randomized controlled trials with modifiable determinants were included. Data extraction and risk of bias assessments were conducted by two independent researchers. Where data could be pooled, we performed Robust Bayesian meta-analyses. Heterogeneity, publication bias and certainty of evidence were assessed. Fifteen studies were deemed eligible to be included. Thirty-seven unique determinants within four settings were identified–school, family, school with family/home, and community with(out) other settings. Ninety-eight percent of determinants belonged to individual/interpersonal determinant categories. Narratively, intervention effects on student perception of teachers’ behaviour (school), self-management, perceived barriers, external motivation, exercise intention, parental modeling on SB (school with family/home) and MVPA expectations (community) were weak to strong, however, corresponding PAB/SB change was not evident. There were negligible effects for all other determinants and the corresponding PAB/SB. Meta-analyses on self-efficacy, attitude, subjective norm and parental practice and PAB/SB in two settings showed weak to strong evidence *against* intervention effect, while the effect on knowledge could not be determined. Similarly, publication bias and heterogeneity for most analyses could not be ascertained. We found no concrete evidence of association between the modifiable determinants and self-report PAB/SB in any settings. This is presumably due to intervention ineffectiveness. Design of future interventions should consider to follow the systems-based approach and identify determinants unique to the context of a setting, including policy and environmental determinants.

## Introduction

Globally, about 18% (over 34 million) children and adolescents are overweight or obese–a 10-fold increase from 40 years ago [1]. Physical inactivity has been identified as one of the main risk factors whereby two-thirds of children and adolescents are insufficiently active, despite the widely recognized benefits of physical activity [2]. Evidence shows that inactive children are likely to become inactive adults [3, 4], and it is projected that the healthcare burden of physical inactivity-related non-communicable diseases will cost INT$520 billion annually between 2020–2030 if the physical inactivity pandemic continues [5].

Over the past three decades, the number of physical activity behaviour (PAB) and sedentary behaviour (SB) interventions targeting childhood inactivity has seen an upward surge [6]. PA is defined as any movement produced by skeletal muscles that involve the energy expenditure of >1.5 metabolic equivalents of tasks (METs) whereas ≤1.5 METs while awake is considered as SB [7]. Conclusions about the effectiveness of interventions for school-aged children from recent systematic reviews have been mixed [8–12]. Typically, these interventions aim to manipulate factors associated with PAB and/or SB, hence these factors are also considered as determinants as their causal associations with PAB or SB are assumed [13]. Not only should determinants be evidence-based, but they should also be modifiable to the extent that can enact behaviour change [14]. An array of determinants relevant to the youth population within the European context has been previously identified by experts of PAB and SB [14, 15]. Based on the socio-ecological model [16], the majority of the identified PAB/SB determinants (approximately 55%) considered to be highly modifiable and have the largest effect on PAB/SB, belong to the individual and interpersonal level, such as attitude, support of peers/family and TV exposure [14, 15]. However, there have been mixed findings on the extent to which interventions that target these determinants are associated with changes in PAB/SB [17–19]. A lack of understanding of which determinants have significantly contributed to changes in PAB/SB has hampered progress in physical activity promotion across the lifespan [20]. Given this state of uncertainty, the DEterminants of Physical Activity in SettingS (DE-PASS) consortium was formed with an aim to identify key determinants effective in promoting PAB and reducing SB, and crucially, translatable at the policy level to accelerate research-policy collaborations in addressing the physical inactivity pandemic.

Several factors may have contributed to the mixed findings regarding the association between the modifiable determinants and PAB/SB. First and foremost, the context within a setting in which the determinants operate is seldom considered [21]. Interventions are often complex for many reasons, including but not limited to the stakeholders involved and their motivation, the physical and psychological capacity for (long-term) implementation and the prevailing PAB/SB practice where the interventions are implemented. As such, the extent to which these factors may influence the modifiable determinants may vary considerably in different settings [22–24]. For example, results of realist reviews of interventions for children showed that in the family setting, physical activity knowledge combined with parental reinforcement was an important determinant unique to that setting, whereas parental restrictions on PAB, as a determinant, hampered the effect of school-based interventions [21, 22]. Another factor that warrants attention is the age groups included in reviews targeting youth, where interventions involving children and adolescents were examined collectively [10, 12, 14]. The developmental journey from childhood to adolescence sees notable changes and adaptations in individuals’ environmental, physical and psychological conditions, all of which define the individuals’ context [25]. For example, while self-efficacy was found to be a common modifiable PAB determinant for children and adolescents, intention appears to be unique to children and perceived behaviour control and planning are unique to adolescents [26, 27]. Given the above considerations, the current systematic review will examine PAB/SB determinants in interventions from different settings, targeting children aged 5–12 years only.

To address a main objective of DE-PASS of generating a Best Evidence STatement (BESt) with regards to the key modifiable determinants for youth PAB/SB from existing best evidence, the current review aimed to examine interventions that target PAB and/or SB using the randomized controlled trial (RCT) and controlled trial (CT) designs. While RCTs are considered the gold standard in intervention design, CTs could be a viable alternative when randomization is challenging due to factors such as participants’ or stakeholders’ preference. This review also focused on self-report PAB/SB measures only, while other planned systematic reviews addressing the same DE-PASS objective will focus on device-based measures, as the discrepancy in measurement is evidenced [28–30]. Therefore, the aims of this systematic review were three-fold–i) to identify the modifiable determinants that have been targeted in PAB/SB interventions in different settings, ii) to evaluate the extent to which these determinants have been modified, and iii) to investigate their association with self-report PAB/SB in school-aged children.

## Methods

This review is one of the five planned systematic reviews conducted under the same deliverable (youth focus) within the DE-PASS consortium. Workshops for all members involved in the review activities were conducted to ensure mutual understanding of the eligibility criteria and the practice in study screening, data extraction, risk of bias assessments, and the use of Covidence, an online systematic review platform (www.covidence.org).

### Study design

This systematic review was conducted following the Preferred Reporting Items for Systematic Reviews and Meta-Analysis Protocols (PRISMA-P) guidelines (S1 Checklist) [31]. The study protocol was prospectively registered in PROSPERO (CRD42021282874).

### Search strategy

The current study applied the same search strategy for all the five systematic reviews under the same deliverable (youth focus) within DE-PASS. A search was conducted on MEDLINE, PsycINFO, Web of Science, Sport Discus and Cochrane Central Register of Controlled Trials for literature from 2010 up to July, 2023. We considered publications from 2010 because this was when WHO published the first global PA guidelines [32]. For the full search strategies and terms, please refer to the published study protocol http://dx.doi.org/10.1136/bmjopen-2021-059202 [33].

### Eligibility criteria

#### Population

We included children aged 5–12 years (inclusive) without known medical conditions that would hinder habitual PAB, such as spina bifida and arthritis.

#### Interventions

Interventions targeting PAB/SB in children using self-report, and that i) had measured modifiable determinants at ≥2 time points (pre-/post-measurements), and ii) had measured the PAB/SB outcomes at ≥2 time points (pre-/post-measurements) were included.

#### Comparator

All studies included a control group receiving no intervention, or a comparator group receiving an alternative intervention matched to the experimental conditions.

#### Outcomes

This review included two types of outcomes–modifiable determinants and self-report PAB/SB, as the intervention effect on both were examined separately. We assessed whether an outcome is qualified as a determinant by the theoretical underpinning or the context of the interventions. For example, if an intervention explicitly aimed to reduce body weight in order to promote PAB, body weight status was considered a determinant. If, however, body weight was clearly considered as an outcome without specifying its mechanistic influence on PAB in the context of the intervention, and given no other modifiable determinants were included, the study was excluded. For studies with both self-report and device-based PAB/SB measurements, only the former was analyzed in this review.

#### Study design

Interventions that followed RCT or CT designs of any duration and follow-up period, and within any settings, were analyzed. Peer-reviewed studies in any language were considered. For studies that did not provide relevant information for eligibility assessment or for data extraction, authors were contacted. These studies were excluded if the requested information was not obtained.

#### Study selection and data extraction

At the initial screening, Endnote x9 was used to remove duplicates and non-peer-reviewed literature. The final identified studies were transferred to Covidence for title/abstract/full-text screening and data extraction. Extracted data included sample characteristics, study characteristics, settings, theoretical basis of the interventions, measurements of PAB/SB and determinants as well as their measurement properties. Study screening and data extraction were completed by deliverable members of DE-PASS in pairs independently. Conflicts were solved by discussion or with a third member.

### Quality assessment

With regards to risk of bias assessments, we used the Cochrane Risk of Bias Tool for Randomized Trials version 2 (RoB2.0) [34] and Risk of Bias in Non-Randomized Studies of Intervention (ROBINS-I) [35] for RCTs and CTs respectively. The ‘Bias in the measurement of outcome’ domain was assessed for both outcomes of interest separately, namely determinant(s) and PAB/SB. Two independent reviewers assessed the risk of bias. A third reviewer was consulted if consensus could not be reached. The assessment plots were generated by the robvis tool [36]. To assess the certainty of evidence, three authors (FCML, AM, KN) followed the Grading of Recommendations Assessment, Development and Evaluation (GRADE) approach to evaluate all studies included in the meta-analyses [37]. GRADE includes five criteria–risk of bias, inconsistency, indirectness, imprecision and publication bias. The level of certainty ranges from high to very low, depending on the extent to which the true effect is considered similar to the estimated effect.

### Statistical analysis

All determinants were categorised based on the socio-ecological model [16]. For determinants with multiple indicators (e.g., different parental practices to minimize screen time), either the composite score was calculated (see S1 File) [38], or a total score provided by the authors was used. Conceptually similar determinants were grouped for analysis where possible.

For studies that reported multiple PAB/SB outcomes, the one that most reflected total daily PAB/SB was used (e.g., habitual MVPA). Composite scores of SB were calculated for SB outcomes that could be combined to reflect habitual SB (e.g., total daily screen time and total daily computer use).

To account for the possible co-variance of individual scores within each composite score, sensitivity analyses were conducted where different correlation coefficients were applied to the formula by Borenstein (2011), to test if effect size might change substantially [38]. Where results at multiple time points were recorded during an intervention, only results at post-intervention were considered (pre-post effect). Additionally, if more than one time-point follow-ups were reported, e.g., after three weeks, one month, and three months, the latest time-point results were used to reflect a longer-term effect (pre-follow-up effect).

Standard mean difference and standard error for changes in determinants and PAB/SB outcomes (from baseline to follow up) were calculated for each included study where possible (see S1 File for details). Individual studies were first inspected for corresponding determinant and PAB/SB changes. For determinants and PAB/SB that could be pooled for meta-analysis, Robust Bayesian meta-analysis (RoBMA) was conducted in JASP 0.16.4 [39, 40], which uses the RoBMA R package [41] and Markov Chain Monte Carlo algorithms via JAGS [42]. We used only random-effects part of the RoBMA model ensemble with the default prior distributions, resulting in 18 included models (detailed RoBMA specification can be found in [43]). We used Bayes factor (BF_01_) to measure evidence of the absence of an effect over the presence of an effect. The same criteria were also applied to publication bias assessment. While the Bayes factor is a continuous measure of strength of evidence, we used the following rule of thumb to aid interpretation: 1<BF_01_<3 = weak evidence (i.e., presence or absence of an effect cannot be ascertained), 3<BF_01_<10 = moderate evidence, BF_01_>10 = strong evidence for the null [44]. When evidence for the alternative was considered, the Bayes factor was simply inverted (e.g., BF_01_ = → BF_10_ = 3, which implies weak evidence *for* an effect). Cohen’s *d* with 95% credible interval (CI) was also reported. Cohen’s *d* ≥ 0.2 (small effect), ≥ 0.5 (moderate effect), ≥ 0.8 (strong effect) [45]. The degree of heterogeneity was assessed by the between-study standard deviation τ. Studies that could not be included in the meta-analyses were reported narratively. For readers unfamiliar with RoBMA, we supplemented the results of the corresponding indicators, including effect size (95% CI), heterogeneity and publication bias, using classical frequentist analysis with random effects meta-analysis and Vevea and Hedges (1995) selection model for publication bias correction (see S2 File) [46]. All intervention settings and outcomes (PAB vs SB, and habitual vs non-habitual PAB were analysed separately. RCTs vs CTs were also examined separately for the purpose of GRADE. The effect of interventions on determinants was analysed regardless of their PAB or SB outcomes.

## Results

### Study selection

**Fig 1:**
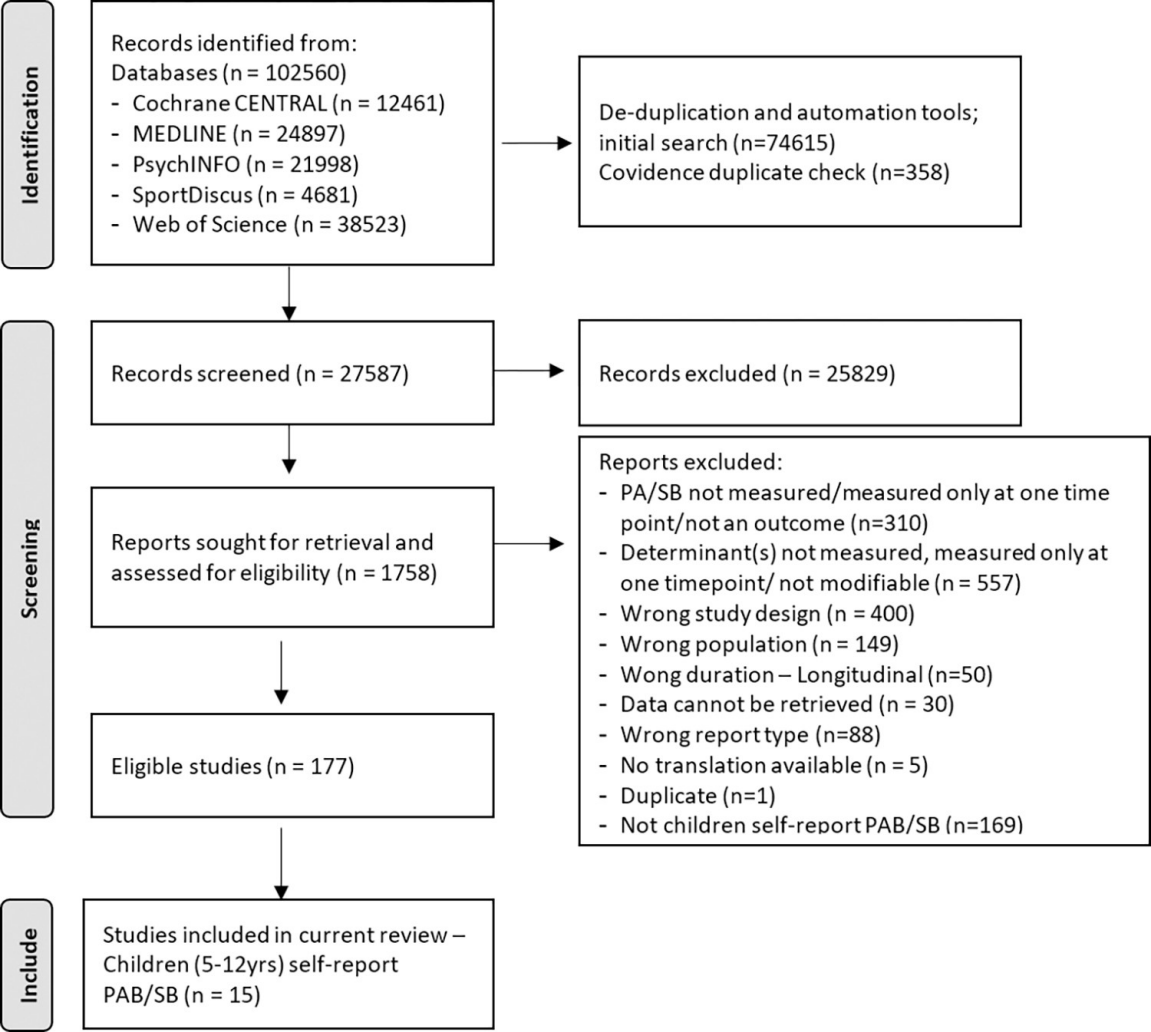
PRISMA flow diagram.

After removing duplicates, 27,581 studies were subject to title and abstract screening. Screening resulted in 1,762 full texts to be assessed for eligibility. Out of the 184 eligible studies, 15 were deemed relevant to the current review (self-report PAB/SB measurement with children 5-12yrs) (Fig 1). Excluded studies can be found in S1 Data, and full extracted data with data extractor information and data extraction date can be found in S2 Data.

### Study characteristics

Table 1 shows the study characteristics and participant demographics of the 15 studies included in this review (10 RCTs, five CTs), totalling 13,107 participants. The settings were determined based on where the interventions were delivered. Four settings were identified from this pool–school only, family only, school with family/home and community with/without other settings (e.g., family/home, school). Interventions that took place in the home environment but without involving parents explicitly, and those that targeted parental involvement, were all classified as family/home setting. Thirty-seven distinct determinants were identified– 25 were individual (psychological) determinants, seven were interpersonal (psychological) determinants, two were individual (behavioural) determinants, another two were interpersonal (behavioural) determinants and one was institutional determinant. Further study characteristics and evidence synthesis for each setting are provided in Table 1. Effect size and 95% CI for each determinant and PAB/SB outcome by settings can be found in S1 Appendix).

**Table 1:** Study characteristics based on settings.

Study identifier		Country	Intervention descriptions	Intervention duration	Follow-up duration	Comparison group(s)	Theoretical basis	Study design	Sample characteristics at baseline	Type of PAB/SB and measurement	Determinant (measurement)	Determinant category based on socio-ecological model [16]
Boyle-Holmes, 2010 [47]		USA	PE curriculum focusing on motor skills progression. 51 lessons per grade, 2 days/week, 30mins/day	2 years	n/a	Control–Received usual PE curriculum	Not stated	CT	n = 1,464 Age: 8-12y Mean age: 9.8 Sex: not reported	Habitual PA–Self-administered Physical Activity Checklist (SAPAS, Sallis et al., 1996)	Motor skill specific self-efficacy (performance measures, van Beurden et al., 2003)	Individual–psychological
											Perception of PA competence (Perceived Physical Activity Competence Scale, Harter, 1982)	Individual–psychological
Wang, 2017 [51]		China	A video game-based intervention to promote healthy eating and PA (Diab). Diab consists of 9 episodes, played in 2x40-min sessions or 1x90min sessions.	8–10 weeks	8–10 weeks	Control–Received general diet and PA information as usual	Social Cognitive Theory, Self-Determination Theory, Elaboration-Likelihood Model	CT	n = 179 Age: 8-12y Sex: 42.5% girls	Habitual PA–PAQ-C at post-intervention* and follow-up* (Wang et al., 2016)	PA self-efficacy at post- intervention* (PA Self-Efficacy scale; Jago et al., 2009)	Individual–psychological
											PA motivation–autonomous and controlled motivation (SDT-based 16-item scale, Deci & Ryan, 2017)	Individual–psychological
											PA preferences (Self-Administered Physical Activity Checklist (SAPAC), Sallis et al., 1996)	Individual–psychological
Gråstén, 2019 [48]		Finland	PE teachers were educated to provide a task-involving climate to promote PA in 26x90-120-min practical sessions over 2 years. School environment was also adapted to promote PA autonomy. All participants received 90mins of PE per week.	12 months	n/a	Control–Received national PE curriculum	Achievement Goal Theory, Social Ecological Model	CT	n = 661 Age: 11-13y Mean age (sd): 12.14 (.31) Sex: 51% girls	Habitual PA—Health Behavior in School-aged Children Research Protocol (HBSC), (Currie et al., 2012)	PE enjoyment (Soini et al., 2006)	Individual–psychological
Gabriel, 2011 [49]		USA	A 12-week curriculum to promote PA and positive youth development, getting participants to engage in 5k runs (Girls on the Run)	12 weeks	5 months	Control–Received no intervention	Not stated	CT	n = 877 Age: ≤9 - ≥11 Sex: 100% girls	Habitual PA–PAQ-C (Crocker et al., 1995)	Physical Activity commitment at follow-up** (Neilson, 1986)	Individual–psychological
Lonsdale, 2019 [50]		Australia	A teacher professional learning intervention, delivered partially via the internet, designed to maximize opportunities for students to be active during PE lessons and enhance adolescents’ motivation towards PE and PA (Activity and Motivation in Physical Education; AMPED)	7–8 months	14–15 months	Control–Received no intervention	Self-Determination Theory	Clustered RCT	n = 1,421 Mean age (sd): 12.93 (0.54) Sex: 43% girls	Leisure time MVPA–Adolescent Physical Activity Measures (Prochaska et al., 2001)	Motivation towards Leisure time Physical Activity–Amotivation, autonomous motivation, controlled motivation (Behavioral Regulation in Exercise Questionnaire; Markland & Tobin, 2004)	Individual–psychological
											Motivation towards PE–Amotivation, autonomous motivation, controlled motivation (Behavioral Regulation in Exercise Questionnaire; Markland & Tobin, 2004)	Individual–psychological
											Needs Satisfaction in PE–Autonomy need, competence need, relatedness need (multiple scales, Standage et al., 2003; McAuley et al., 1989; Richer & Vallerand, 1998)	Individual–psychological
											Student Perceptions of PE Teacher Behavior—Controlling behaviour at follow-up* (Controlling Coach Behaviors Scale, CCBS; Bartholomew et al., 2010) Student Perceptions of PE Teacher Behavior—Supportive behaviour (Belmont et al., 1988)	Interpersonal–psychological
Maddison, 2014 [52]		New Zealand	Parents were provided with information (in face-to-face meeting, monthly newsletters and a dedicated website) regarding SB and how to reduce SB. Children also received an activity pack with alternatives to SB activities (SWITCH; Screen-Time Weight-loss Intervention Targeting Children at Home)	24 weeks	n/a	Control–Received no intervention	Social Cognitive Theory, Behavioural Economics Theory	RCT	n = 251 Age: 9-12y Sex: 43% girls	SB–Total (min/day) PAB–MET/day (Multimedia Activity Recall for Children and Adolescents; MARCA) (Ridley et al., 2006)	Perceived enjoyment of SB (Salmon et al., 2003)	Individual—psychological
											Perceived enjoyment of PA Physical Activity Enjoyment Scale (Motl et al., 2001)	Individual—psychological
											Primary caregiver total PA–IPAC LF (Booth et al., 2003)	Interpersonal—behavioural
Pearce, 2019 [53]	Australia		School -based training on PA-related knowledge (benefits of PA, goal-setting and PA at home) (10x1hr), home-based activity booklet and parent workshops on healthy eating and PA.	10 weeks	10 weeks	Control–Received no intervention	Social Cognitive Theory	CT	n = 147 Age: 9-13y Sex: 59% girls	Habitual PA—PAQ-C (Crocker et al., 1997)	Self-management at follow-up* (6 items Dishman et al., 2005)	Individual–psychological
											Perceived barriers to PA at post-intervention* (9 items Dishman et al., 2005)	Individual–psychological
											Outcome expectancy value of PA (9 items, Dishman et al., 2010)	Individual–psychological
											Enjoyment of PA (6 items, Motl et al., 2001)	Individual–psychological
											Self-efficacy (8 items, Dishman et al., 2002)	Individual–psychological
											Social support–home (Sallis et al., 2002)	Interpersonal—psychological
											School support -school at follow-up** (Sallis et al., 2002)	Interpersonal—psychological
Quaresma, 2014 [55]	Portugal		Health and weight educational program (PESSOA) with homework completed with parents to raise awareness of child’s behaviour and its assessment.	52 weeks	n/a	Control–Received no intervention	Self-Determination Theory	Clustered RCT	n = 617 Mean age (sd): 10.42 (1.09)	Habitual PA–PAQ (Telama et al., 1997)	Peer support (Ommundsen et al., 2008)	Interpersonal—psychological
											Teacher support (Ommundsen et al.,2008)	Interpersonal—psychological
											Parental social support* (Ommundsen et al.,2008)	Interpersonal—psychological
											Parental encouragement (Ommundsen et al.,2008)	Interpersonal—psychological
											Amotivation (BREQ-2 Palmeira et al., 2007) External motivation* (BREQ-2 Palmeira et al., 2007) Introjected motivation (BREQ-2 Palmeira et al., 2007) Identified motivation (BREQ-2 Palmeira et al., 2007) Intrinsic motivation (BREQ-2 Palmeira et al., 2007)	Individual—psychological
Zhang, 2020 [56]	China		The intervention encompassed 5 theoretical courses on benefits of PA, disadvantages of SB and sport skills, plus 3 outdoor basketball matches (45mins/session, 1 session/week). Also involved parents to make exercise plans and log exercise every week.	8 weeks	n/a	Control–Received no intervention	Theory of Planned Behaviour, Social Cognitive Theory	RCT	n = 51 Mean age (sd): 12 (0.3) Sex: 47.1% girls	Habitual PA—International Physical Activity Rating Scale (Craig et al., 2003)	Self-efficacy*** (no reference to theoretical constructs, not the validity of questionnaires, but offer alpha from current study)	Individual—psychological
											Outcome expectancy (no reference to theoretical constructs, not the validity of questionnaires, but offer alpha from current study)	Individual—psychological
											Exercise Attitude (Francis et al., 2004)	Individual—psychological
											Subjective norm (Francis et al., 2004)	Interpersonal—psychological
											Perceived behavioural control (Francis et al., 2004)	Individual—psychological
											Exercise Intention*** (Francis et al., 2004)	Individual—psychological
Bergh, 2014 [54]	Norway		Intervention consisted of lessons on PA, screen and dietary behaviour at school. Tailored feedback was provided on how to change screen behaviour. Parents received fact sheet on parental regulation of screen behaviour.	20 months	n/a	Control–Received no intervention	Social Ecological Model, Social Cognitive Theory	Clustered RCT	n = 1,418 Mean age (sd): 11.2 (0.26) Sex: 40.4% girls	SB—Screen behaviour (TV and computer/electronic games use; hr/day) (no reference provided)	Perceived parental regulation TV-viewing (Hardy et al., 2006)	Interpersonal—psychological
											Perceived parental regulation computer/electronic games use (Hardy et al., 2006)	Interpersonal—psychological
Vik, 2016 [57]	Belgium, Germany, Greece, Hungary, Norway		Lessons at school on SB, goal setting to reduce SB and how to do it at home (45mins/week). Assignments to be completed at home or at school. Six newsletters to parents on personalized messages from children and homework tasks to be completed by the children, and sometimes with parents (UP4FUN, part of ENERGY).	6 weeks	n/a	Control–Received no intervention	Model of Planned Promotion for Population Health, Socio-Ecological Model	Clustered RCT	n = 3,147 Age: 10-12y Mean age: 11.2y Sex: 51.2% girls	SB–Sedentary time (TV/DVD viewing, PC/Games console use; hr/day) (van Stralen et al., 2011, Singh et al., 2011)	Self-efficacy (van Stralen et al.,2011)	Individual—psychological
											Attitude (van Stralen et al.,2011)	Individual—psychological
											Preferences/liking (van Stralen et al., 2011)	Individual—psychological
											Automaticity (van Stralen et al.,2011)	Individual—psychological
											Awareness (van Stralen et al.,2011)	Individual—psychological
											Knowledge (van Stralen et al.,2011)	Individual—psychological
											Parental practices (van Stralen et al.,2011)	Interpersonal—behavioural
											Parental modeling* (van Stralen et al.,2011)	Interpersonal—psychological
											Parental subjective norm (van Stralen et al.,2011)	Interpersonal—psychological
											Availability of TV/DVD/PC consoles (van Stralen et al., 2011)	Institutional
Salmon, 2010 [59]	Australia		The intervention consisted of 6 lessons (1 lesson/week) on PA and health, identifying TV viewing practice and alternative activities (Switch-2-Activity).	7 weeks	n/a	Control–Wait-list	Social Cognitive Theory, Behavioural Choice Theory	Clustered RCT	n = 957 Age: 9-12y Mean age (sd): 10.3 (0.62) Sex: 58% girls	SB–Screen-based entertainment (min/day) (Salmon et al., 2005) Habitual MVPA (min/dady) (Telford et al., 2004)	Self-efficacy in reducing SB and PA (Saunders et al., 1997)	Individual—psychological
											PA self-efficacy (Saunders et al., 1997)	
											TV viewing style (Salmon et al., 2006)	Individual—behavioural
Moitra, 2021 [58]	India		The intervention comprised of weekly lessons on healthy eating and PA (50-60mins/week). To encourage engagement, a workbook and interactive educational materials were provided. Parents also attended 3 monthly sessions on healthy eating and PA, and how to incorporate small changes in their children’s lifestyle (Health Eating and Activity Program for Schoolchildren, HEAPS).	12 weeks	n/a	Control–Received no intervention	Health Belief Model	Clustered RCT	n = 498 Age: 10-12y Sex: 48.1% girls	Habitual MVPA*, habitual SB–Questionnaire psychometrics tested within the study, partially presented	PAB/SB-related knowledge **- Questionnaire psychometrics tested within the study, but not presented	Individual—psychological
**Setting–Community with or without other settings**												
Branscum, 2013 [60]	USA		Using comic books to educate about PAB/SB in an after-school program. One lesson/week, 30mins/session (knowledge-based; Comics for Health)	4 weeks	3 months	Active control–theory-based covering SCT concepts	Social Cognitive Theory	RCT	n = 71 Age: 8-11y Sex: 46.5% girls	Habitual PA (mins) Sedentary time (mins) at post-intervention** (School PA and nutrition questionnaire) (Thiagarajah et al., 2008)	MVPA/SB self-efficacy—Promoting healthy lifestyles survey (Sharma et al., 2005)	Individual—psychological
											MVPA/SB expectations at follow-up*—Promoting healthy lifestyles survey (Sharma et al., 2005) (reliability questionable)	Individual—psychological
											MVPA/SB self-control—Promoting healthy lifestyles survey (Sharma et al., 2005) (reliability questionable)	Individual—psychological
Christiansen, 2014 [61]	Denmark		Active travel to school policies implemented, including encouraging parents to take active transport to schools, traffic education at school and advocating improvement of safety in the environment for active school transport (SPACE—for physical activity).	2 years	n/a	Control–Received no intervention	Not stated	Clustered RCT	n = 1,348 Mean age (sd): 12.6 (0.63) Sex: 49% girls	Transportation PA—% Active trips to school (Toftager et al., 2011) (unspecific about psychometric properties)	Parents encourage cycling to school (Toftager et al., 2011) (unspecific about psychometric properties)	Interpersonal—psychological
											Perceived safe route to school (Toftager et al., 2011) (unspecific about psychometric properties)	Individual—psychological
											Positive attitude towards bicycling (no reference on instrument used; psychometrics unknown)	Individual—psychological

*Note*: Under ‘type of PAB/SB and measurement’ and ‘determinant’ columns–text in **bold** denotes an intervention effect: small intervention effect (*d*≥ 0.2 <0.5)*, moderate effect (*d*≥ 0.5 < 0.8)** and strong effect (*d*≥ 0.8)*** based on Cohen’s *d*.

### (I) School setting

Five studies–one RCT and four CTs–with sample sizes ranging from 179 to 1,464 were identified. Intervention duration ranged from eight weeks to two years, and follow-up periods from the end of the interventions ranged from eight weeks to 15 months. Four interventions targeted changes in PE curricula and/or PE teacher training [47–50], and one intervention implemented a video game-based program at schools [48]. Three interventions were theory informed [48, 50, 51]. Four studies measured habitual PA [47–49, 51], and one study measured leisure time PA [50], using validated instruments (see Table 1).

#### Study outcomes—PAB/SB and determinants

*RCT*. Six conceptually different determinants were targeted in the RCT, of which four belonged to the individual (psychological) category and two belonged to the interpersonal (psychological category) [50]. The psychometric properties of all determinant measurements were referenced (Table 1). There was no significant change in determinants at immediate post-intervention and at follow-up (*d*’s ranged from -0.17 to 0.28), except a small effect on students’ perception of teachers’ controlling behaviour at follow-up (*d* = 0.25, 95%CI 0.12 to 0.37), indicating teacher’s behaviour was perceived to be more controlling which is against what the intervention aimed to achieve. Additionally, there was non-significant effect on PAB (*d* = -0.02).

**Fig 2:**
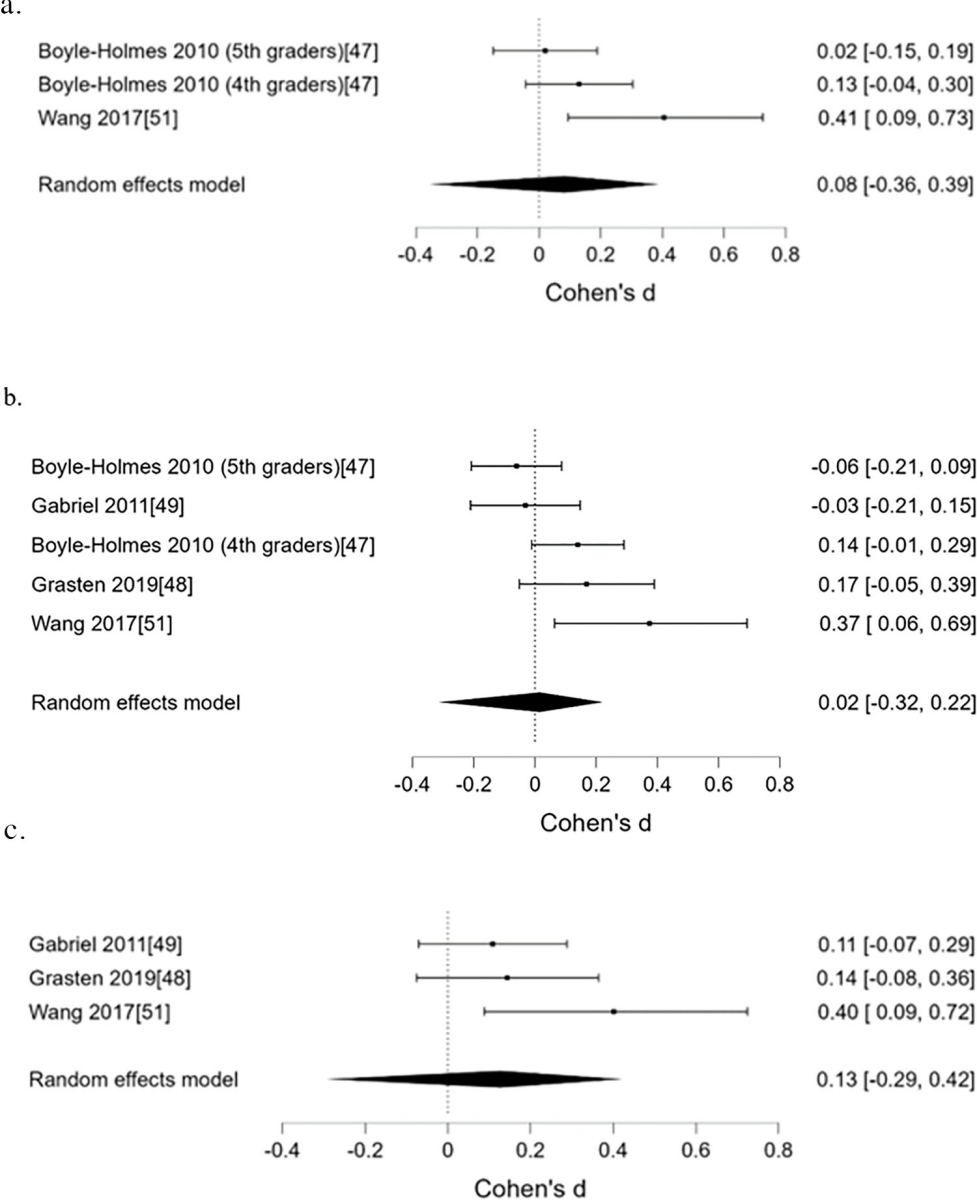
Forest plots depicting intervention effect on (a) self-efficacy, (b) overall PAB pre-/post- effect and (c) overall PAB pre-/follow-up effect in CTs under school setting.

*CTs*. Five distinct individual (psychological) determinants were targeted in all four studies. The psychometric properties of all determinant measurements were referenced (see Table 1). Only self-efficacy from one study showed a small effect at post-intervention (*d* = 0.40; 95% CI 0.09 to 0.73) [51], however, when it was pooled in a meta-analysis with another study [44], there was moderate evidence against an effect on self-efficacy (Table 2; Fig 2A). Narratively, there was moderate intervention effect on commitment to PA (*d* = 0.68; 95% CI 0.50 to 0.87) [49]. However, none of the determinants that could only be analysed narratively reported notable intervention effect (*d*’s ranged from -.17 to 0.28; for determinants that showed small effects, the 95% CI’s crossed the estimate threshold).

**Table 2:** Results of the meta-analyses under the school setting and the corresponding heterogeneity and publication bias assessments. The effect size estimates for meta-analyses and heterogeneity are expressed in *d* (95%CI) and τ respectively.

	Effect size estimates	BF_01_
**Self-efficacy for PAB (2 CTs) (Fig 2A)**	0.08 (-0.39, 0.40)	4.85*
Heterogeneity (τ)	0.15 (0.04, 0.45)	-
Publication bias	-	0.48
**PAB (4 CTs) (pre-post) (Fig 2B)**	0.00 (-0.41, 0.23)	8.08*
Heterogeneity (τ)	0.12 (0.03, 0.29)	-
Publication bias	-	0.46
**PAB (2 CTs) (pre-follow up) (Fig 2C)**	0.15 (-0.39, 0.60)	2.91
Heterogeneity (τ)	0.21 (0.04, 0.73)	-
Publication bias	-	0.63

Note

*denotes moderate evidence

**denotes strong evidence for absence of an effect/ publication bias.

For the individual study that showed a small effect on self-efficacy at post intervention, there was a corresponding small effect on PAB at the same time point [51]. For the study that measured commitment to PA, there was no corresponding effect on PAB. When all CTs were pooled for meta-analysis, there was moderate evidence against an effect on PAB at post-intervention (Fig 2B), however, there was insufficient evidence to suggest presence or absence of an effect on PAB at follow-up, or publication bias (Table 2; Fig 2C). Heterogeneity for all meta-analyses seem small, but due to limited number of studies in each meta-analysis, the degree of heterogeneity is highly uncertain.

### Quality assessment

**Fig 3:**
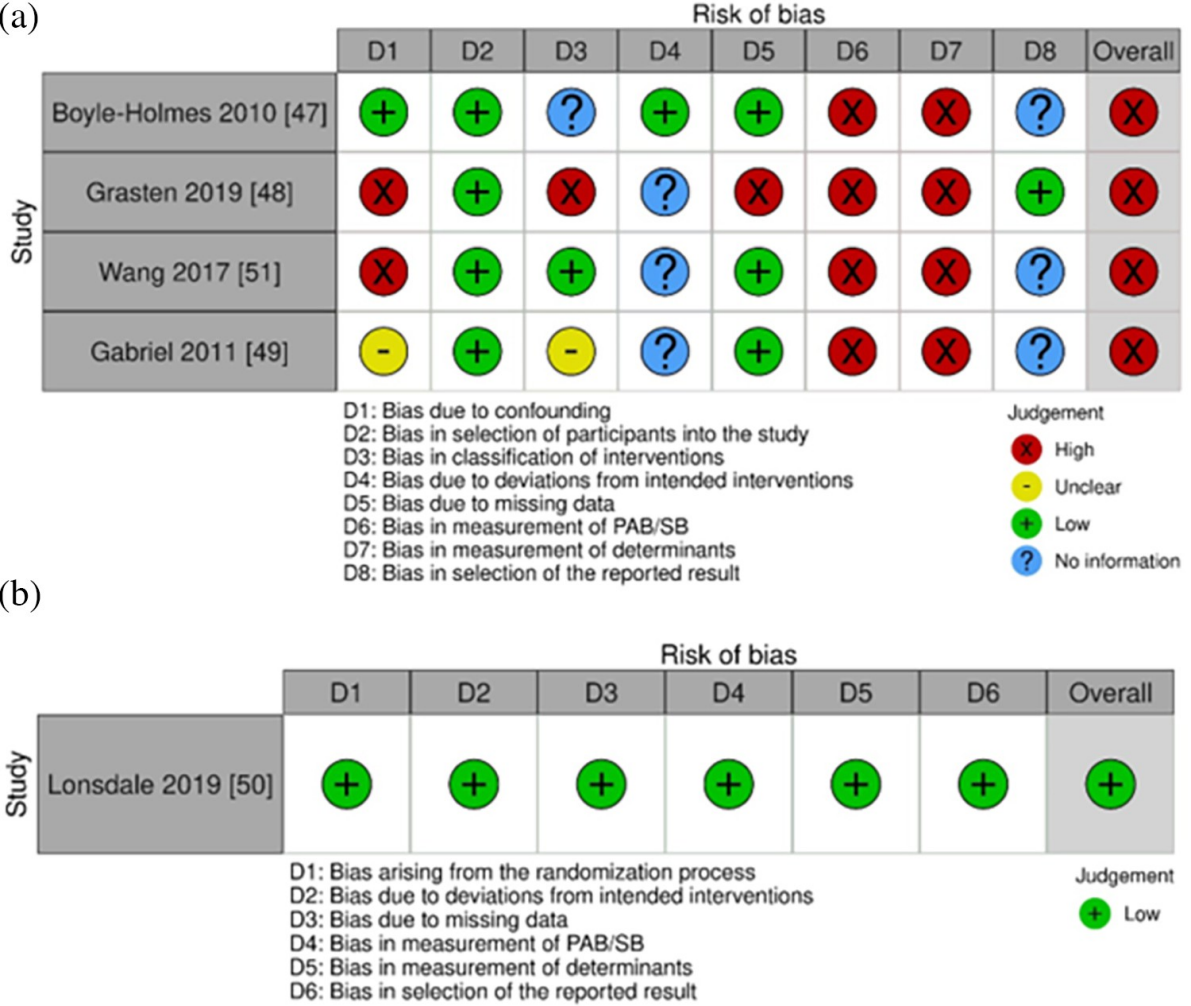
Risk of bias assessments of CTs using (a) Robins-I and risk of bias assessments of the RCT using (b) RoB2.0 in school setting.

For the four CTs, they were all deemed high risk of bias overall. Notable contributors to the judgement were three domains—domain 1 (bias due to confounding), domains 6 and 7 (bias due to measurement of outcomes–PAB/SB and determinants respectively). Judgement for domain 1 primarily stemmed from the fact that not all pre-defined confounders were accounted for in all studies, while judgement for domains 6 and 7 was because participants were unlikely to be blinded in most interventions involving self-report measurements (PAB/SB and determinants) (Fig 3A). Nonetheless, the one RCT in this setting explicitly mentioned blinding of the participants and researchers (Fig 3B) [50].

#### Certainty of evidence and intervention effect

We conducted GRADE for the meta-analysis of self-efficacy in two CTs (Table 3A) [47, 51], and the meta-analysis with PAB as an outcome in four CTs at post-intervention [47–49, 51]and the two CTs at follow-up [49, 51] (Table 3B). The certainty of evidence was high for the absence of intervention effect on PAB at post-intervention, whereas for the other two meta-analyses, the certainty of evidence was deemed low mainly due to imprecision of effect estimate.

**Table 3:** a. Overview of quality of evidence (GRADE) and intervention effect on self-efficacy for two CTs in school setting. **b.** Overview of quality of evidence (GRADE) and intervention effect on physical activity for 4 CTs in school setting.

Certainty assessment								№ of participants at baseline		Intervention effect (*d*, 95%CI) Heterogeneity (τ)	Certainty	Importance
**a)**												
**№ of studies**	**Authors, year**	**Study design**	**(1)**	**(2)**	**(3)**	**(4)**	**(5)**	**Intervention**	**No intervention**			
**Outcome: Self-efficacy (intervention duration: 8 weeks to 64 weeks)**												
2	Boyle-Holmes et al. (2010) [47] Wang et al. (2017) [51]	Controlled trials	NS	NS	NS	VS^A^	none	855	788	*d* = 0.07; 95%CI = -0.36, 0.39 τ - CBD	⨁⨁◯◯ Low	IMPORTANT
**b)**												
**Outcome: Physical Activity (intervention duration: 8 weeks to 64 weeks)**												
4	Boyle-Holmes et al. (2010) [47] Gabriel et al. (2011) [49] Gråstén & Yli-Piipari (2019) [48] Wang et al. (2017) [51]	Controlled trials	NS	NS	NS	NS	none	1,407	1,774	*d* = 0.00; 95%CI = -0.41, 0.23 τ - CBD	⨁⨁⨁⨁ High	CRITICAL
**Outcome: Physical Activity (follow-up: 10 weeks to 5 months)**												
2	Gabriel et al. (2011) [49] Wang et al. (2017) [51]	Controlled trials	NS	NS	NS	VS^A^	none	685	371	*d* = 0.15; 95%CI = -0.39, 0.60 τ - CBD	⨁⨁◯◯ Low	IMPORTANT

Note

As three risk of bias domains for CTs (bias due to confounding and outcome measurement bias) and two domains for RCTs (outcome measurement bias) are almost inevitable in the nature of the interventions conducted, it was decided that they should be treated more leniently in GRADE; (1) = risk of bias, (2) = inconsistency, (3) = indirectness, (4) = imprecision, (5) = other considerations; *d* = Cohen’s *d*, 95%CI = 95% confidence interval; CBD = Cannot be determined as there is little evidence of presence or absence of heterogeneity; NS = Not serious; VS^A^ = very serious concern with a relatively wide 95%CI.

### (II) Family/home setting

**Fig 4:**
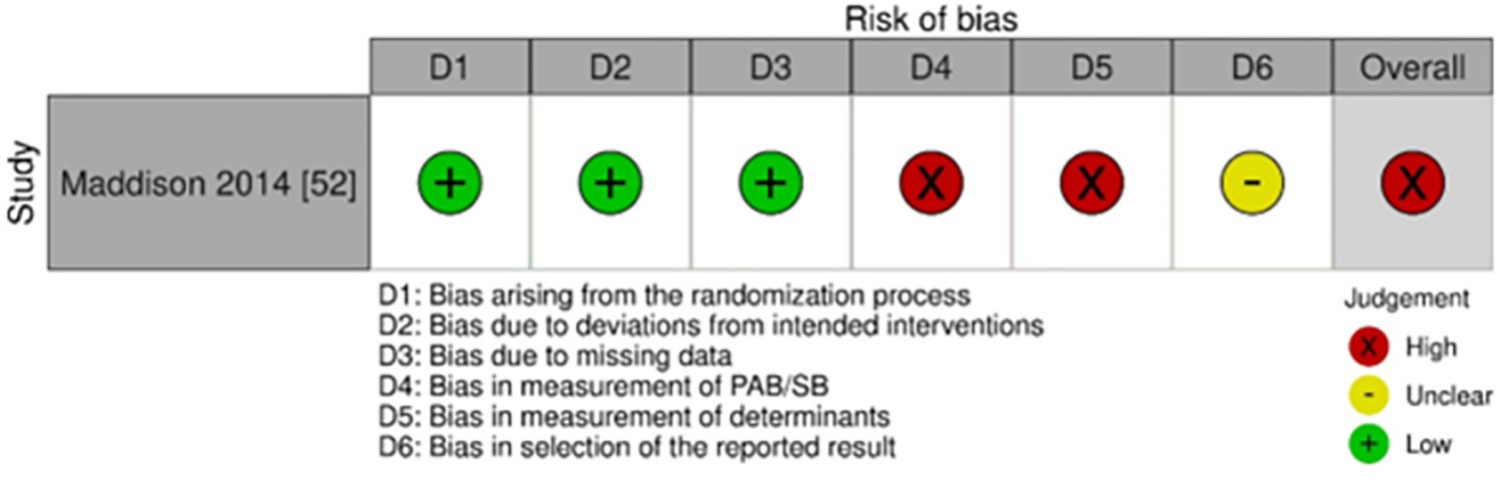
Risk of bias assessment of an RCT using RoB2.0 in family/home setting.

Only one intervention was conducted in the family/home setting (see Table 1) [52]. It was a theory-based 24-week RCT with no follow-up assessment, and both habitual PAB and SB were examined (n = 251). Three determinants were measured using validated instruments–two belonged to individual (psychological) and one belonged to interpersonal (behavioural) categories. No significant intervention effects were reported for all outcomes (*d*’s ranged from -0.17 to 0.10). The risk of bias was deemed high due to bias in the measurement of outcomes (domain 4 and 5 –PAB/SB and determinants respectively) (Fig 4).

### (III) School with family/home settings

We identified seven studies of which one was a CT [53], and all interventions were theory-informed (see Table 1). Sample sizes ranged from 51 to 3,147. Intervention period ranged from six weeks to 20 months, and only 1 study included follow-up (10 weeks post-intervention) [53]. Four interventions indirectly involved parents in the form of homework completion with children or remote knowledge provision [54–57], two interventions actively involved parents in workshops or information sessions [53, 58], and one intervention relied on children adhering to the home intervention [59]. Habitual PAB [53, 55, 56] and habitual MVPA [53] were measured in the respective studies, and SB was measured in four studies [54, 57–59].

#### Study outcomes—PAB/SB and determinants

*RCTs*. Twenty-three conceptually different determinants were targeted in these interventions (see Table 1). Fifteen determinants belonged to the individual (psychological) category, one belongs to individual (behavioural) category, five belonged to the interpersonal (psychological) category, one belongs to the interpersonal (behavioural) category and one belonged to the institutional category.

Out of all RCTs, determinants that showed positive effects were–i) parental support (*d* = 0.24, 95%CI (0.09, 0.40)) [52],external motivation (*d* = -0.23, 95%CI -0.38 to -0.07) [55] and parental modelling on SB (*d* = 0.25, 95%CI 0.18 to 0.32) [57]–small effect; ii) knowledge (*d* = 0.50, 95%CI 0.31 to 0.68) [58]–moderate effect, and iii) self-efficacy (*d* = 0.90, 95%CI (0.31, 1.57)) and exercise intention (*d* = 0.87, 95%CI 0.30 to 1.45) [56]–strong effect (Table 1). Other determinants showed non-significant intervention effect (*d*’s ranged from -0.44 to 0.52; for determinants that showed small to moderate effects, the 95% CI’s crossed the estimate threshold) [54–59].

**Fig 5:**
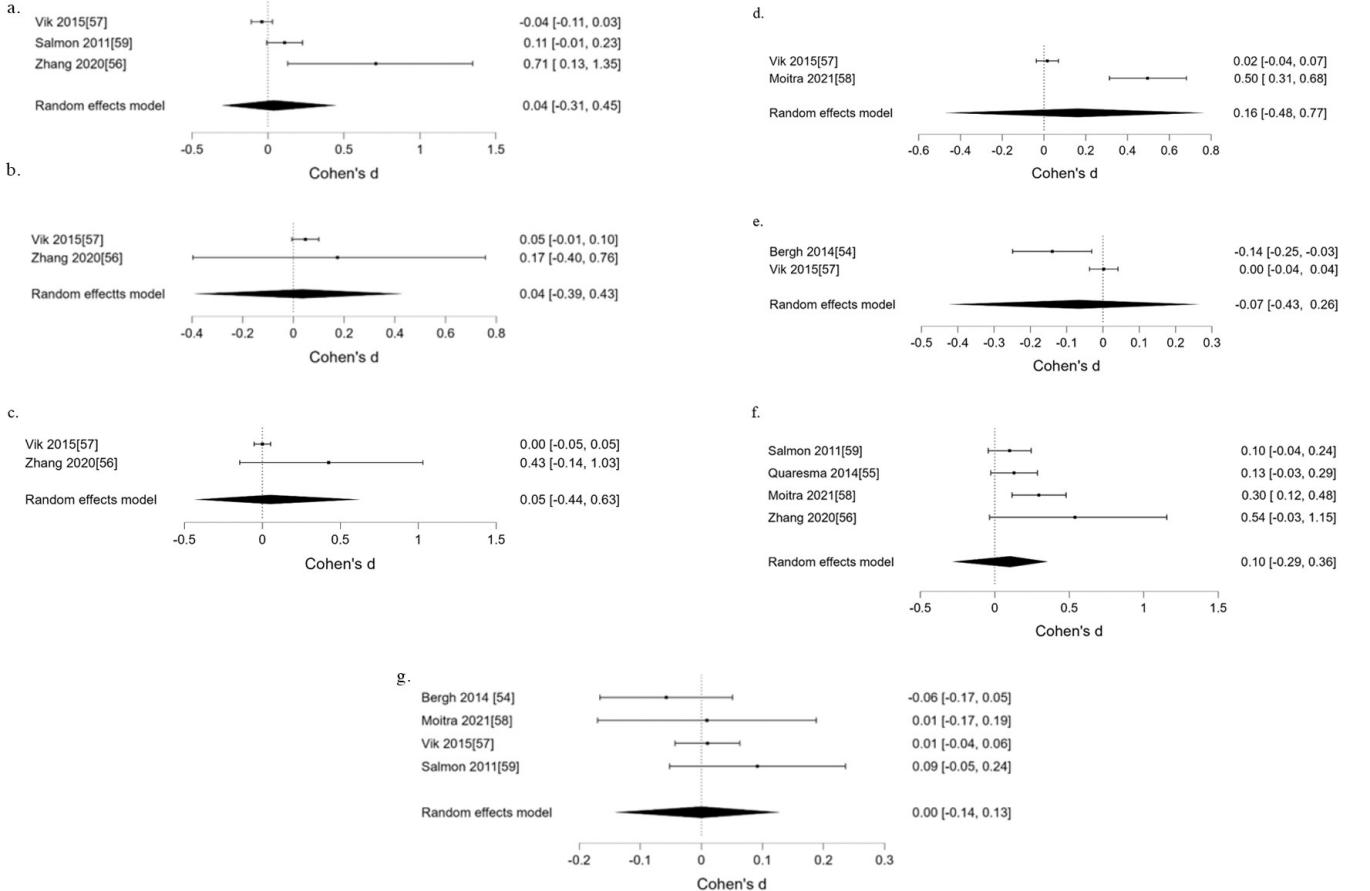
Intervention effects on (a) self-efficacy in studies targeting PAB and/or SB; (b) attitude in studies targeting PAB and/or SB; (c) subjective norm in studies targeting PAB and/or SB; (d) knowledge in studies targeting SB; (e) parental practice in SB regulation in studies targeting SB; (f) PAB at post-intervention; (g) SB at post-intervention, in RCTs under school and family/home settings.

Five determinants were targeted in more than one study–self-efficacy [56, 57, 59], attitude [56, 57], subjective norm [56, 57], knowledge [56, 58] and parental practice in SB regulation [54, 57], hence we conducted a meta-analysis for each (Fig 5A–5E). Results of meta-analyses showed moderate evidence against an effect on self-efficacy, attitude, subjective norm and parental practice (Table 4). There is moderate evidence of presence of publication bias for the meta-analysis on self-efficacy (BF_10_ = 5.88). Evidence for presence or absence of an effect on knowledge and publication bias for other meta-analyses cannot be determined.

**Table 4:** Results of the meta-analyses under the school with family/home setting, with the corresponding heterogeneity and publication bias assessments. The effect size estimates for meta-analyses and heterogeneity are expressed in *d* (95%CI) and τ respectively.

	Effect size estimates	BF_01_
**Self-efficacy for PAB/SB (3 RCTs) (Fig 5A)**	0.05 (-0.31, 0.52)	7.63*
Heterogeneity	0.15 (0.03, 0.56)	-
Publication bias	-	0.17*
**Attitude for PAB/SB (2 RCTs) (Fig 5B)**	0.04 (-0.41, 0.49)	7.46*
Heterogeneity	0.14 (0.03, 0.47)	-
Publication bias	-	1.12
**Subjective norm for PAB/SB (2 RCTs) (Fig 5C)**	0.04 (-0.04, 0.57)	7.63*
Heterogeneity	0.15 (0.03, 0.56)	-
Publication bias	-	0.99
**Knowledge for SB (2 RCTs) (Fig 5D)**	0.16 (-0.05, 0.78)	2.89
Heterogeneity	0.32 (0.05, 1.01)	-
Publication bias	-	0.64
**Parental practice for SB (2 RCTs) (Fig 5E)**	-0.06 (-0.43, 0.32)	7.67*
Heterogeneity	0.14 (0.06, 0.46)	-
Publication bias	-	1.79
**PAB (4 RCTs) (pre-/post) (Fig 5F)**	0.10 (-0.29, 0.36)	3.70*
Heterogeneity	0.14 (0.04, 0.38)	-
Publication bias	-	0.13*
**SB (4 RCTs) (pre-/post)(Fig 5G)**	0.00 (-0.14, 0.13)	19.67**
Heterogeneity	0.08 (0.03, 0.19)	-
Publication bias	-	1.98

Note

*denotes moderate evidence

**denotes strong evidence for absence of an effect/heterogeneity/publication bias.

For intervention effect on PAB/SB, meta-analyses showed moderate evidence against an effect on PAB (Fig 5F) and strong evidence against an effect on SB (Fig 5G). There was moderate evidence for publication bias for PAB (BF_10_ = 7.69). In one study included in the meta-analysis for PAB and for knowledge [58], moderate effect on knowledge (the only determinant measured) and small effect on PAB (*d* = 0.30, 95%CI 0.12 to 0.48) [58] was found, however, the psychometrics of knowledge measurement was not referenced. Therefore, despite that some interventions showed promise on narratively analysed determinants (i.e., external motivation [55], exercise intention [56], and parental modelling [57]), corresponding change on the pooled PAB/SB effect is not evident. Again, the magnitude of heterogeneity of all meta-analyses appeared small, but this remains inconclusive due to small number of studies in each meta-analysis (Table 4). Together with other results that did not see any corresponding changes in determinants and PAB/SB, we could only suggest that associations between these determinants and PAB/SB were possible, and that the interventions had not been successful in changing either.

*CT*. The only CT under this setting showed small effect on perceived barriers to PA (*d* = 0.43, 95%CI 0.06 to 0.80) at post-intervention and self-management (*d* = 0.43, 95%CI (0.06, 0.81)) at follow-up. There was a moderate effect on social support from schools at follow-up (*d* = 0.53, 95%CI 0.16 to 0.91). However, PAB notably decreased at post-intervention (*d* = -0.51, 95%CI -0.88 to -0.13) and there was negligible intervention effect on PAB at follow-up (*d* = 0.00) [53].

### Quality assessment

**Fig 6:**
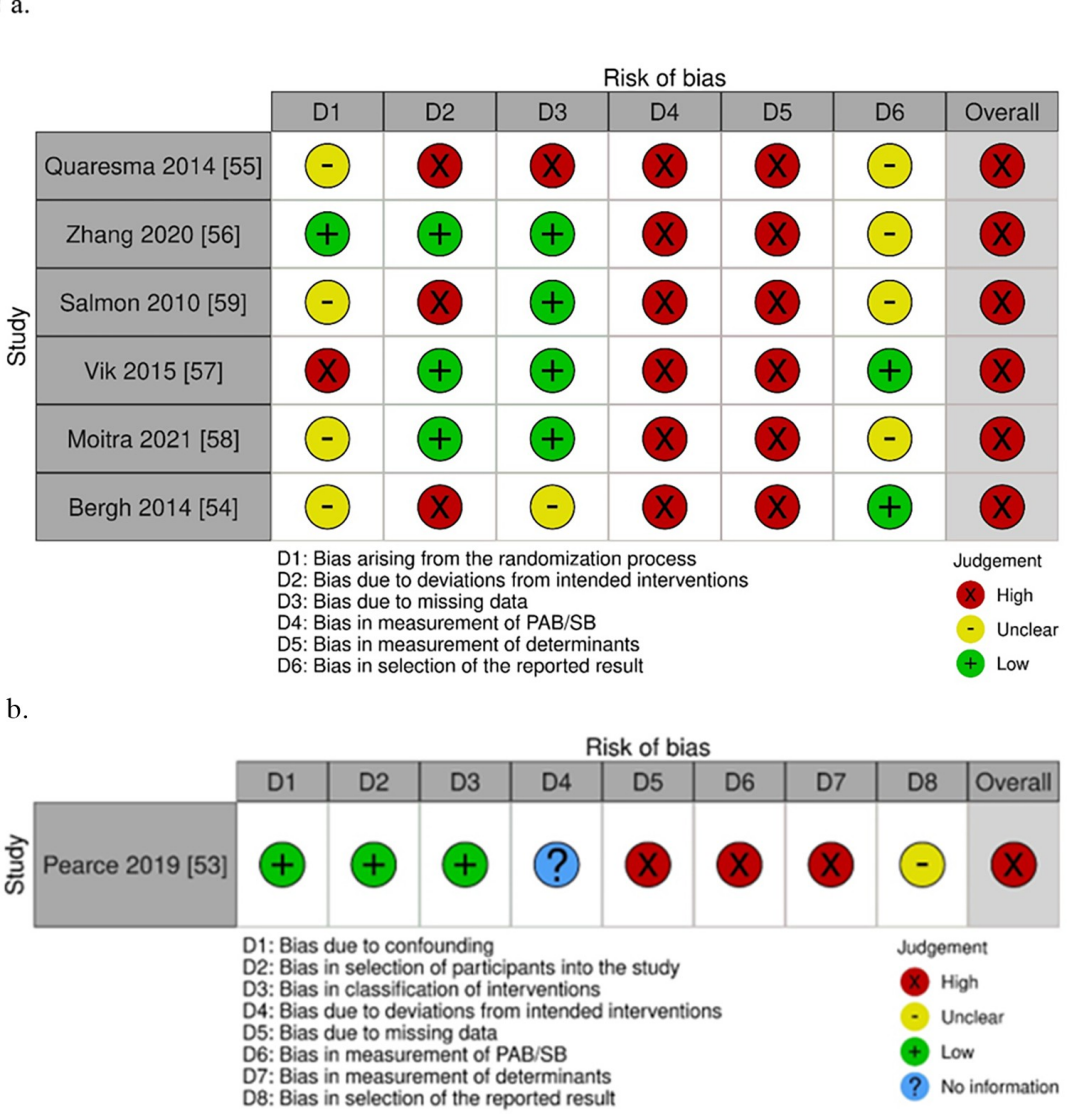
Risk of bias assessments of (a) RCTs and (b) CT in school with family/home settings.

All studies are deemed high risk of bias, mainly due to bias in the measurement of outcomes (PAB/SB and determinants) (Fig 6).

#### Certainty of evidence and intervention effect

We conducted GRADE for the seven meta-analyses on self-efficacy, attitude, subjective norm, knowledge, parental practice, PAB and SB (all RCTs; Table 5A and 5B). The certainty of evidence was downgraded to low or very low for all meta-analyses due to risk of bias and/or imprecision.

**Table 5:** a. Overview of quality of evidence (GRADE) and intervention effect on self-efficacy, attitude, subjective norm, knowledge and parental practice for RCTs in school with family/home setting. **b.** Overview of quality of evidence (GRADE) and intervention effect on physical activity and sedentary behaviour in school with family/home setting.

Certainty assessment								№ of participants at baseline		Intervention effect (*d*, 95%CI) Heterogeneity (τ)	Certainty	Importance
**a)**												
**№ of studies**	**Authors, year**	**Study design**	**(1)**	**(2)**	**(3)**	**(4)**	**(5)**	**Intervention**	**No intervention**			
**Outcome: Self-efficacy of physical activity/sedentary behaviour (intervention duration: 8 weeks to 64 weeks)**												
3	Salmon et al. (2011) [59] Vik et al. (2015) [57] Zhang et al. (2020) [56]	Randomised controlled trials	S^A^	NS	NS	VS^A^	none	2,154	2,179	*d* = 0.04; 95%CI = -0.29, 0.44 τ - CBD	⨁◯◯◯ Very low	CRITICAL
**Outcome: Attitude towards physical activity/sedentary behaviour (intervention duration: 6 weeks to 8 weeks)**												
2	Vik et al. (2015) [57] Zhang et al. (2020) [56]	Randomised controlled trials	NS	NS	NS	VS^A^	none	1,687	1,689	*d* = 0.04; 95%CI = -0.41, 0.49 τ - CBD	⨁⨁◯◯ Low	CRITICAL
**Outcome: Subjective norm towards physical activity/sedentary behaviour (intervention duration: 6 weeks to 8 weeks)**												
2	Vik et al. (2015) [57] Zhang et al. (2020) [56]	Randomised controlled trials	NS	NS	NS	VS^A^	none	1,687	1,689	*d* = 0.16; 95%CI = -0.52, 0.78 τ - 5.35 (based on BF_01_)	⨁⨁◯◯ Low	CRITICAL
**Outcome: Knowledge on sedentary behaviour (intervention duration: 6 weeks to 12 weeks)**												
2	Vik et al. (2015) [57] Moitra et al. (2021) [58]	Randomised controlled trials	NS	VS^B^	NS	VS^A^	none	1,955	1,868	*d* = 0.04; 95%CI = -0.47, 0.57 τ - CBD	⨁◯◯◯ Very low	CRITICAL
**Outcome: Parental practice on sedentary behaviour (intervention duration: 6 weeks to 80 weeks)**												
2	Vik et al. (2015) [57] Bergh et al. (2014) [54]	Randomised controlled trials	S^B^	S^C^	NS	VS^A^	none	2,172	2,571	*d* = -0.06; 95%CI = -0.43, 0.32 τ - CBD	⨁◯◯◯ Very low	CRITICAL
**b)**												
**Outcome: Self-efficacy of physical activity/sedentary behaviour (intervention duration: range 8 weeks to 64 weeks)**												
4	Salmon et al. (2011) Quaresma et al. (2014) Zhang et al. (2020) Moitra et al. (2021)	Randomised controlled trials	S^B^	NS	NS	S^D^	none	1,199	924	*d* = -0.10; 95%CI = -0.29, 0.36 τ - CBD	⨁◯◯◯ Very low	CRITICAL
**Outcome: Sedentary behaviour (intervention duration: range 6 weeks to 80 weeks)**												
4	Salmon et al. (2011) Bergh et al. (2014) Vik et al. (2015) Moitra et al. (2021)	Randomised controlled trials	S^B^	NS	NS	S^D^	none	2,931	3,267	*d* = 0.00; 95%CI = -0.14, 0.13 τ - 11.49 (based on BF_01_)	⨁⨁◯◯ Low	CRITICAL

Note

As three risk of bias domains for CTs (bias due to confounding and outcome measurement bias) and two domains for RCTs (outcome measurement bias) are almost inevitable in the nature of the interventions conducted, it was decided that they should be treated more leniently in GRADE; (1) = risk of bias, (2) = inconsistency, (3) = indirectness, (4) = imprecision, (5) = other considerations; *d* = Cohen’s *d*, 95%CI = 95% confidence interval; CBD = Cannot be determined as there is little evidence of presence or absence of heterogeneity; NS = Not serious; S^A^ = High risk of bias in randomisation, deviation from intended intervention, bias in reporting in three studies, and moderate evidence of the presence of publication bias; S^B^ = A combination of some concerns and high risks in a few domains in the risk of bias assessment; S^C^ = Marginal overlap of 95%CI (not including point estimates); S^D^ = Imprecision mainly comes from one study with lowest weight, 95%CI of overall estimate includes small to moderate effects in both directions; S^E^ = Relatively long tails of CI; Estimates of two studies closer to 0.0, estimates of the other two studies are on either side further away from 0.0 VS^A^ = point estimate is near 0, but 95%CI of overall effect includes small to moderate effect in both directions; VS^B^ = no overlap in 95%CI and very different estimates.

### (IV) Community with/without other settings

Two RCTs were identified (see Table 1). One intervention was conducted in a community setting only with an active control group [60], and one was in the community with family/home and school setting (where parents were indirectly involved in the intervention and active policy/environmental adaptation was in place) [61]. Sample sizes ranged from 71 to 1,348. Interventions lasted from four weeks to two years, and follow-up periods ranged from three to six months. Only one intervention was theory-informed [60]. One study measured habitual PAB and SB [60] and one targeted transportation PA [61], only the former measurement instruments was referenced.

#### Study outcomes—determinants and PAB/SB

Within the two studies, six determinants were targeted–five were individual (psychological) and the other one was interpersonal (psychological). The measurements, and measurement properties, of three determinants within one study were unspecific [56] (see Table 1).

Due to difference in the type of PAB/SB measured [60, 62], PAB/SB outcomes can only be analysed descriptively. The community-based intervention showed a moderate post-intervention effect on SB (*d* = -0.74, 95%CI -1.22 to -0.26), with trivial effects on all determinants (*d*’s ranged from 0.18–0.33; for determinants that showed small effects, the 95%CI’s crossed the threshold). However, the small effect on MVPA/SB expectations at follow-up (*d* = 0.39, 95%CI 0.04 to 0.75) did not see any changes to PAB or SB [60]. For the other study, there were only trivial effects for perceived safe route to school, parental encouragement for cycling to school, attitude towards cycling and % active trips to school (*d*’s ranged from -0.14 to 0.14) [61].

### Quality assessment

**Fig 7:**
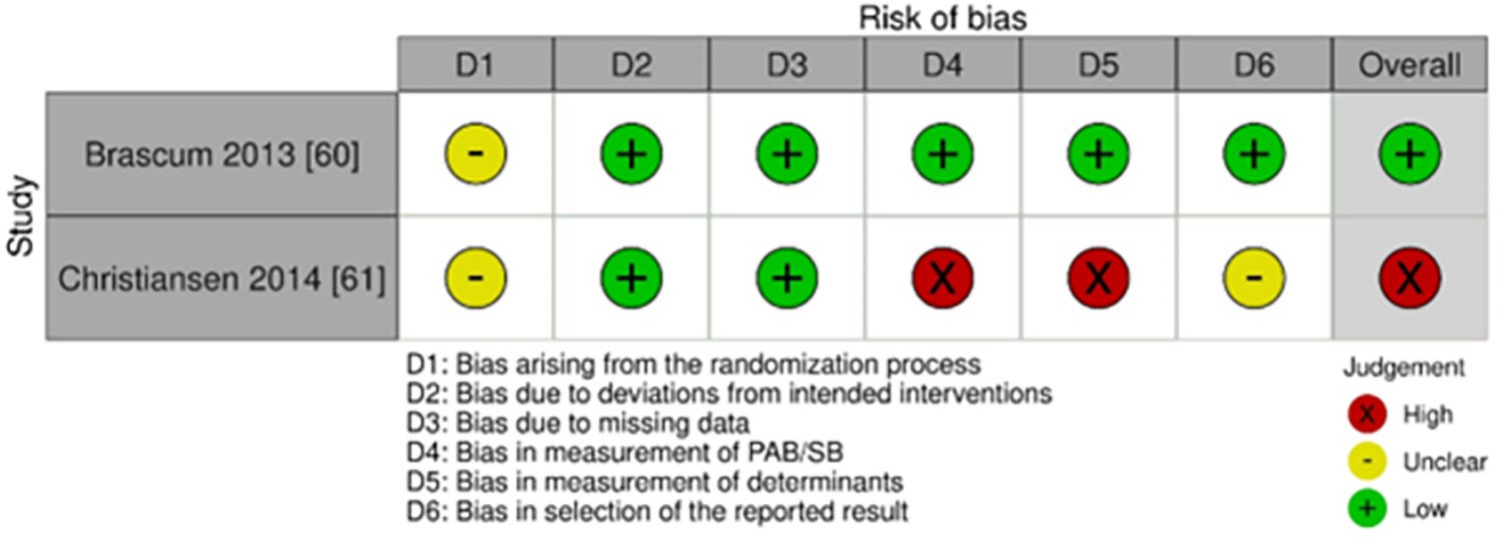
Risk of bias assessment of studies that conducted RCTs in a community setting alone or with other settings (family/home and/or school).

Similar to other studies included in this review, the main reason for the overall high-risk decision on study bias was due to measurement bias [61]. However, the study with an active control group could contribute to blinding of participants, as such, the study was deemed low risk (Fig 7).

### Sensitivity analyses

Sensitivity analyses were performed for studies that required estimations of composite scores. No difference in the effect sizes was detected when *r* was set at 0.2, 0.5 and 0.8.

## Discussion

The main aims of this review were to identify modifiable determinants that have been targeted in interventions that followed the RCT/CT designs and to assess their association with self-report PAB/SB in children in different settings. To our knowledge, it is the first study to apply Robust Bayesian meta-analyses to examine the effects of interventions on modifiable determinants, and to infer the associations between the determinants and PAB/SB where possible. Out of the 37 distinct determinants targeted across all settings, 68% were individual (psychological) determinants, 5% were individual (behavioural) determinants, 20% were interpersonal (psychological) determinants, 5% were interpersonal (behavioural) determinants and there was only one institutional determinant. Common determinants across settings were self-efficacy, family support, school support, peer support, motivation based on self-determination theory, PA enjoyment, caregivers’ PA, perceived competence and attitude. Of all determinants, only six can be pooled for meta-analyses by settings. PA/SB self-efficacy was targeted in the *school* setting (CTs) and *school with family/home* setting (RCTs), and there was moderate evidence *against* an intervention effect. Attitude, subjective norm and parental practice under *school with family/home* setting (RCTs) also showed moderate evidence *against* an effect, while the strength of evidence for knowledge cannot be determined. This is surprising as some of these determinants have been widely targeted in PAB/SB interventions [17, 19, 24].

Regarding PAB/SB, results of the meta-analyses showed moderate evidence of absence of post-intervention effect on PAB (CTs) in the *school setting* and moderate-strong evidence *against* post-intervention effect on PAB and SB (RCTs) in the *school with family/home setting*. The certainty of evidence was either low or very low, except for the absence of intervention effect on PAB in the *school* setting (high certainty). Taken together, the lack of intervention effects on the modifiable determinants might have contributed to unsuccessful PAB/SB change in children.

Considering all studies that showed no corresponding changes between determinants and PAB/SB, we could only conclude that associations between these determinants and PAB/SB were possible, as no change in determinants would not lead to any change in PAB/SB given the assumed association between the targeted determinants and PAB/SB [13]. However, concrete evidence of the associations was not found (i.e. moderate-large effect sizes on both determinants and PAB/SB). Interestingly, we found changes in some determinants but corresponding positive changes in PAB/SB were not evident, namely, perceived barriers to PA at post-intervention [53], self-management and school support at follow-up [53], parental social support [55], external motivation [55], exercise intention [55] and parental modeling in SB [57] (under school and family/home settings) as well as MVPA/SB expectations at follow-up [60] (under community setting). Future research should carefully consider if and how these determinants should be targeted in interventions.

The majority of interventions in the current review were theory-based—as advocated by behaviour change researchers [62]—common theories used being the self-determination theory, goal achievement theory, social cognitive theory and theory of planned behaviour.

However, the intervention effect on determinants and PAB/SB was not significant across settings, despite that most determinants and PAB/SB measurements were evidenced to be psychometrically sound. In fact, our results echoed previous findings on the weak association between some self-determination theory tenets and PAB in the youth population [63]. However, contrary to the results of a recent umbrella reviews of PAB/SB interventions in children, we did not find family support to be associated with behaviour change [64], nor intention or self-efficacy from earlier reviews [26, 27]. Nonetheless, direct comparisons with existing systematic reviews should be cautioned, as the inclusion criteria of different reviews and the analytic strategies are likely to differ. Additionally, the efficacy of theory-based PAB interventions could be compromised by the methodological weakness of the included studies [65], which is potentially applicable to the current review. Whilst every included study inevitably has its limitations, crucially, our results highlighted a bigger picture problem on how the physical inactivity problem is understood, whether it is an individual-level or a population-level issue. Two main factors may have contributed to the failure in changing the determinants and/or (associated) PAB/SB. First and foremost, behaviour change theories that advocate individual-level change solely (including in the context of interpersonal determinants) have long been criticized for their overestimation of people’s self-regulatory ability [66]. Through these theories, individual and interpersonal determinants are derived. A study of Cochrane reviews, from 1993 to 2019, investigating the effect of RCTs on obesity in the youth population shows that consistently, about 57% of interventions target individual and interpersonal determinants only [67]. However, over-reliance on individual agency may have led to failure of policies in tackling the obesity crisis [68] and exacerbated health inequity [69]. This also begs the question–are these determinants as modifiable, or as effective in changing PAB/SB as researchers thought? [14, 15] Perhaps changes in policy and the environment will facilitate change in individual and interpersonal determinants, which will in turn enact the desired behaviour [22]. Such consideration is imperative as different geographical regions have their own PA policies and environmental concerns in place which are likely to affect population-level PAB/SB differently. While there is growing emphasis on targeting policy and environmental determinants through understanding the interactions between actors and determinants within a system (e.g. priority of education policy; a systems-based approach), their changes are challenging to quantify [70]. Nonetheless, realist synthesis can help address the mechanistic associations between the determinants, and enhance our understanding of what works for who, how and in what context [71]. The spirit of realist synthesis can also contain the common problem with heterogeneity in PAB/SB interventions [8, 72]. As such, researchers and public health practitioners should involve stakeholders in developing intervention content specific to a setting that is unique to their needs and political/physical environment [73]. While the systems-based approach might compromise the internal validity of uniform individual-centered interventions, the resulting interventions might see a higher level of buy-in, adherence and ultimately, effectiveness [74].

Another potential explanation for the largely ineffective interventions could be due to an oversight in relatively unconscious motivation that hinders behaviour change [75]. Based on the COM-B model that encapsulates the main ingredients for successful behaviour change [62], all interventions included in this review have targeted individuals’ psychological and/or physical capability (C), have provided social and/or physical opportunities (O), and reflective motivation (M) (referring to the individual/interpersonal psychological determinants), but arguably, automatic motivation to disengage from behaviour change is overlooked. Important to note is that PAB/SB interventions target inactive individuals who are likely to favour being sedentary over being active at a behavioural level [75]. However, PAB promotion seldom considers such inherent resistance to behaviour change [76]. Currently, research into this dual process in behaviour change is largely experimental, so the need for this area of research to be incorporated in applied settings is urgently called for [13]. Not only will such effort benefit intervention design, but also health messaging in the promotion of PAB.

Some limitations of the current review warrant attention. First, this review was based on self-report PAB/SB which is subject to various types of bias, including but not limited to social desirability and recall bias, based on the PAB/SB tools used in the included studies [77]. However, this is not to say that self-reports are inferior to device-based measurements when its usage is fit for purpose [78]. Additionally, the small number of studies included in all meta-analyses makes it challenging to determine the degree of heterogeneity and publication bias, despite that RoBMA was conceptualized to offset the lack of power [43]. Nevertheless, if followed the frequentist approach, publication bias should only be assessed when there are 10 or more studies in a meta-analysis, which all our meta-analyses fall short on [79]. Besides, adopting the Bayesian approach to meta-analysis has benefited our interpretation of the findings, as it can indicate the strength of evidence of the likelihood of the presence or absence of an effect, unlike the all-or-nothing interpretation from the frequentist approach [44]. Another issue with a small study number within a meta-analysis might have contributed to imprecision in the GRADE process even though the total sample is relatively sizable. This has inevitably impacted our assessment of the certainty of evidence. Regarding the risk of bias assessment, as blinding of participants is inherently challenging, if not impossible, due to ethical considerations, the relevant domains related to outcome assessment were deemed ‘high risk’ for all studies. We have thus examined all domains in the risk of bias assessment in the GRADE process instead of relying on the overall risk. Future interventions should consider including an active control group, so that the status of the intervention group can be more easily masked, and any efforts in blinding participants should be made more explicitly clear. Importantly, due to the lack of mediation analyses in the included studies, the association between the determinants and PAB/SB could only be inferred. Whilst the call for mediation analysis to examine the causal pathways was made more than a decade ago [80], many interventions still do not adopt this analytic approach. A potential reason could be the sheer number of determinants (some more modifiable than others) included in some interventions hinder meaningful mediation analyses [81]. For example, in one of the included studies, there are altogether 44 determinants for PAB/SB, and some of these determinants are conceptually similar (e.g., both determinants ‘parents let child watch TV’ and ‘parents remind child about rules’ can fall under one umbrella determinant ‘parental practice on SB’) [57]. Additionally, contradictory evidence exists in the association between determinants and PAB/SB, and researchers ought to monitor their unconscious bias in selecting the determinants to intervene. Without a clearer understanding of the context through which determinants operate and interact with each other, incorporating even evidence-based determinants into an intervention would not guarantee intervention success. Lastly, due to the restrictions of our eligibility criteria, interventions that implemented policy/environment change (as determinants themselves), but without quantifying the magnitude of change, had been excluded from the review. However, these interventions may provide valuable qualitative information regarding the interactions between different levels of determinants within the socio-ecological model. Future research should also review interventions and real-life public health initiatives that targeted policy and environmental change, to examine the extent to which they can effectively modify individual and interpersonal determinants.

## Conclusion

The current systematic review set out to examine modifiable determinants in interventions following the RCT and CT design that target children and their association with self-report PAB/SB in different settings. However, the lack in intervention effect on determinants and the corresponding PAB/SB in all settings led us to conclude that the associations between any modifiable determinants and PAB/SB remain uncertain. Specifically, almost all modifiable determinants identified belonged to individual or interpersonal categories according to the socio-ecological model. None of the meta-analyses showed evidence for the presence of intervention effect on the determinants and PAB/SB. These results made us question the modifiability of individual and interpersonal determinants in different settings, and whether they would be more modifiable if policy and/or environment conducive to PAB/SB change were in place. Additionally, for determinants that have seen an intervention effect, but without corresponding changes in PAB/SB, if and how they should be targeted in future interventions should be carefully considered. Crucially, to accelerate our understanding of what determinants might work for who and how, and in what settings, realist synthesis should be conducted in order to inform the design of interventions, and interventions should adopt a system-based approach. With more careful consideration of determinants to target in interventions, conducting mediation analysis between determinants and PAB/SB could provide a clearer picture of their causal pathways. Lastly, design of interventions for children should also consider the automatic motivation that hinders behaviour change.

## Supporting information

S1 Checklist PRISMA checklist. (DOC)

S1 File Effect size and composite score calculation. (DOCX)

S2 File Frequentist analysis. Frequentist approach to the meta-analyses.
(DOCX)

S1 Data Excluded studies with reasons for exclusion. (XLSX)

S2 Data Full data extraction with data extractor and date of data extraction. (XLSX)

S1 Appendix Effect sizes and CIs for all outcomes. (DOCX)
