# Supplementary material for: DE-PASS Best Evidence Statement (BESt): Determinants of self-report physical activity and sedentary behaviours in children in settings: A systematic review and meta-analyses
Source: PLoS One. 2024 Nov 25;19(11):e0309890. doi: 10.1371/journal.pone.0309890 (PMC11588252; doi:10.1371/journal.pone.0309890)
Supplement: S1 File — (DOCX) [file pone.0309890.s002.docx]

**1a. Effect size calculation**

Continuous outcomes

When within group change scores (SD) were reported in the primary study, these were used to calculate standardized mean difference (SMD). If measures of variance other than SD were reported in each group, e.g., 95%CI or standard error (SE), these were converted to SD as reported in Cochrane handbook (<https://training.cochrane.org/handbook/current/chapter-06#section-6-5-2-2>).

SMD was then calculated using the Cohen’s d formula:

$$SMD=\frac{\mu_{1}-\mu_{2}}{\sigma_{pooled}}$$

$$\sigma_{pooled}=\sqrt{\frac{{(n}_{1}-1)\sigma_{1}^{2}+ {(n}_{2}-1)\sigma_{2}^{2}}{{(n}_{1}+ n_{2}-2)}}$$

Standard error (SE) of SMD was calculated as:

$${SE}_{SMD}=\sqrt{\frac{n_{1}+ n_{2}}{n_{1}n_{2}}+\frac{{SMD}^{2}}{2(n_{1}+ n_{2}-2)}}$$

In cases where:

1. sample size was not provided for both groups separately (i.e., only total sample size available), an equal sample size for each group was assumed;
2. within group change scores (SD) were not reported, pre- and post- SD were transformed to SD change as reported in Cochrane handbook (<https://training.cochrane.org/handbook/current/chapter-06#section-6-5-2-8>). Correlation coefficients were arbitrarily set at 0.5 when this information was not provided in the study;
3. only between-group differences were reported with t statistics or *p*-values, these were converted to SD as reported in Cochrane handbook (<https://training.cochrane.org/handbook/current/chapter-06#section-6-5-2-3>);
4. only binary outcomes (%) with CI were reported, the former was treated as the mean.

**1b. Composite score calculation for multiple outcomes (Borenstein et al., 2011)**

Formula for calculating composite score:

$$var\left( \sum_{a=1}^{m} Y_{a} \right)= \sum_{a=1}^{m} V_{a}+ \sum_{a\neq b} \left( r_{ab}\sqrt{V_{a}} \sqrt{V_{b}} \right)$$

where *Y* is the effect size of an outcome, *V* is the variance of *Y* for several variables *a*=1…m, and *r* is the correlation coefficient that indicates the extent to which the outcomes co-vary.
