# Supplementary material for: DE-PASS Best Evidence Statement (BESt): Determinants of self-report physical activity and sedentary behaviours in children in settings: A systematic review and meta-analyses
Source: PLoS One. 2024 Nov 25;19(11):e0309890. doi: 10.1371/journal.pone.0309890 (PMC11588252; doi:10.1371/journal.pone.0309890)
Supplement: S2 File — Frequentist approach to the meta-analyses. (DOCX) [file pone.0309890.s003.docx]

This supplementary file provides the results of the standard mean differences (SMD) with 95% confidence interval (CI) and heterogeneity for the MAs conducted in this review. For effect size, we considered Cohen’s *d* ≥ 0.2 (small effect), ≥ 0.5 (moderate effect), ≥ 0.8 (strong effect) (Cohen, 1988). We assessed heterogeneity by *I^2^*, whereby values of 25%, 50% and 75% indicates low, moderate and high heterogeneity respectively^[[1]](#footnote-1)^. We were unable to test publication bias by funnel plot asymmetry inspection, as none of the MAs included the recommended ≥10 studies^[[2]](#footnote-2)^.

**School setting**

1a.


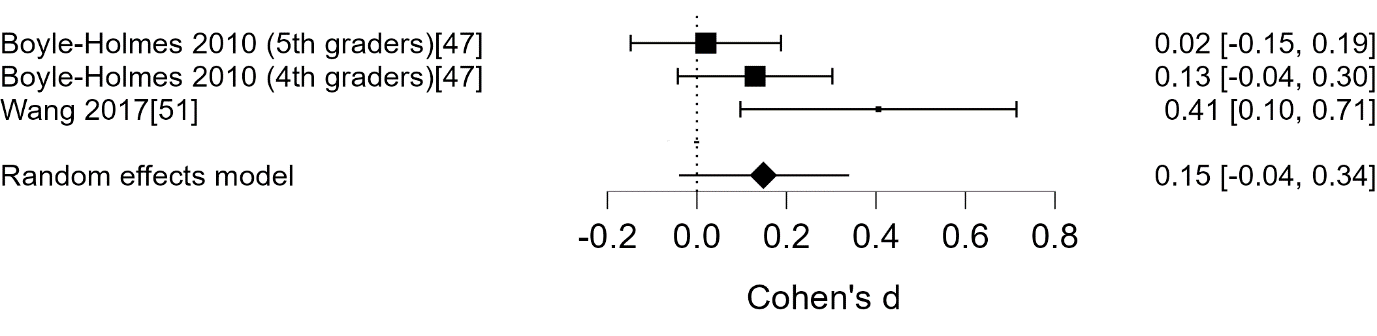


1b.


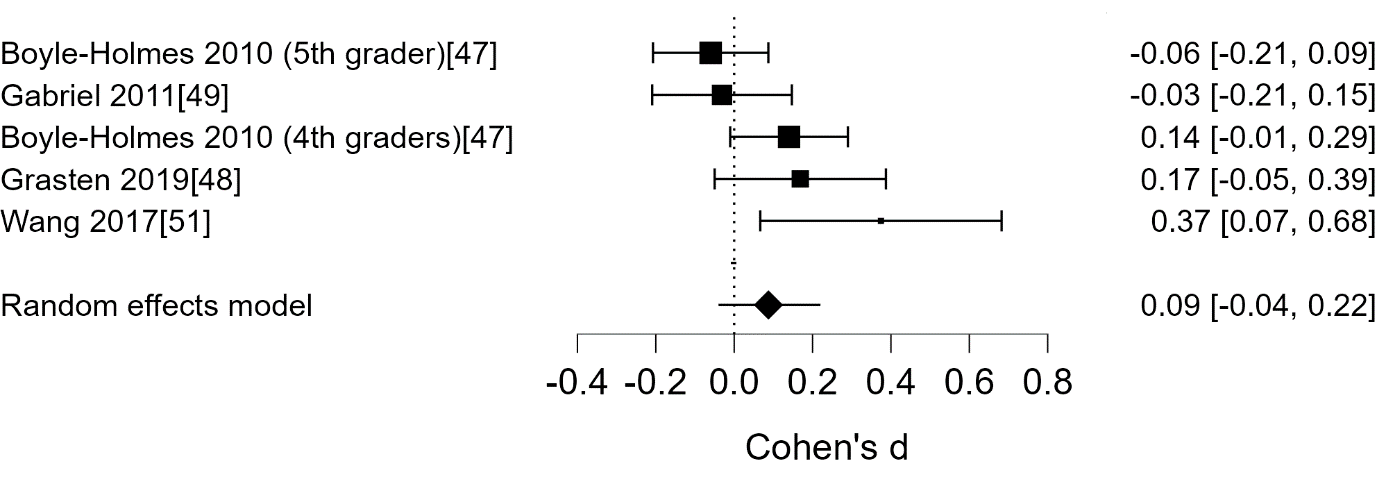


1c.


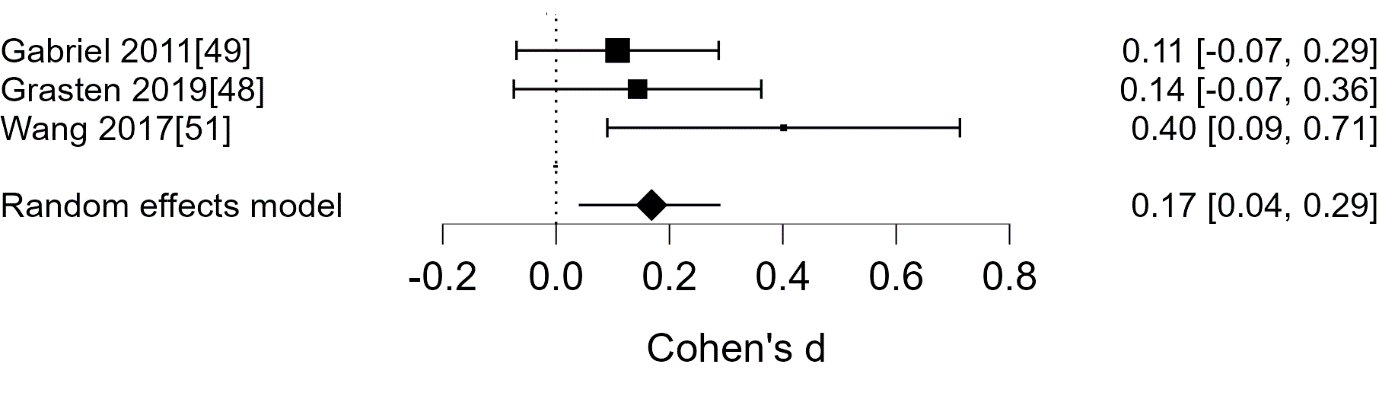


Figure 1. Within school setting only, forest plots showing intervention effect on determinants and PAB in CTs.

(a) Represents post-intervention effect on self-efficacy, *d*=0.15, 95%CI (-0.04, 0,34), *I^2^* = 59.43% (moderate heterogeneity);

(b) Represents overall pre-/post- post-intervention effect on PAB, *d*=0.09, 95%CI (-0.04, 0.22), *I^2^* = 53.54% (moderate heterogeneity).

(c) Represents overall pre-/follow-up effect on PAB, *d*=0.23, 95%CI (-0.06, 0.51), *I^2^* = 60.99% (moderate heterogeneity).

**School with family/home setting**

2a.


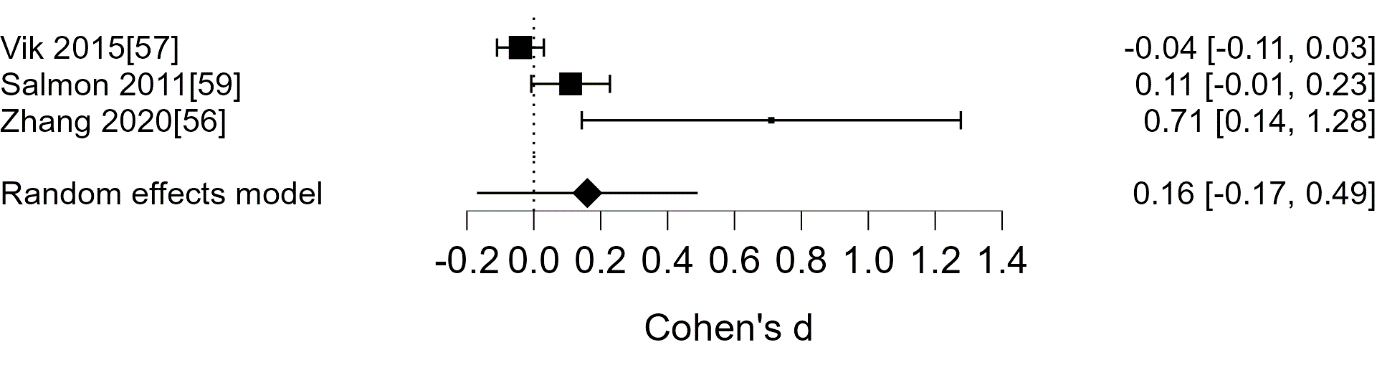


2b.


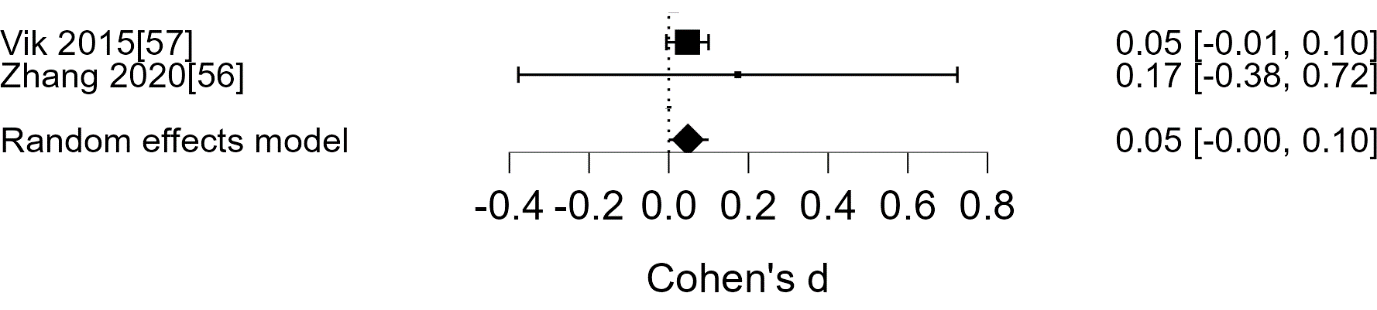


2c.


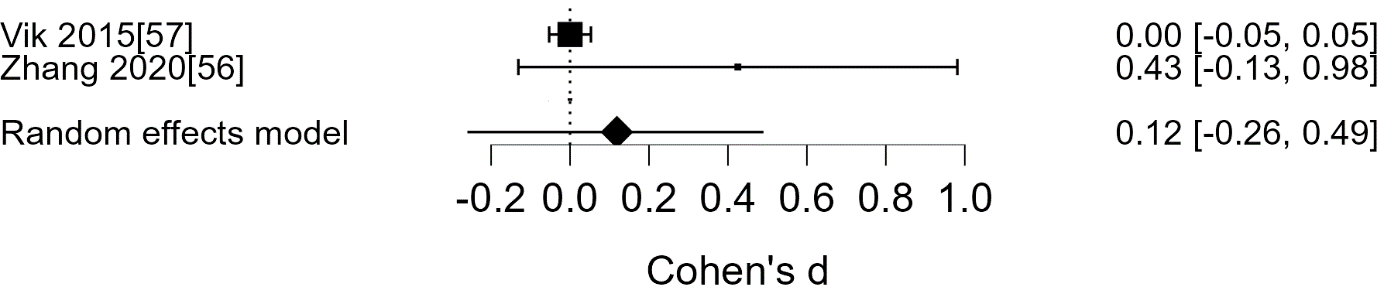


2d.


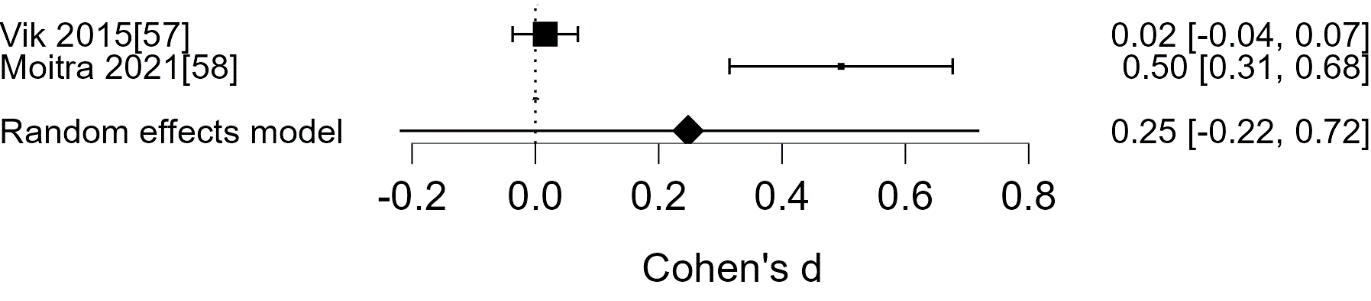


2e.


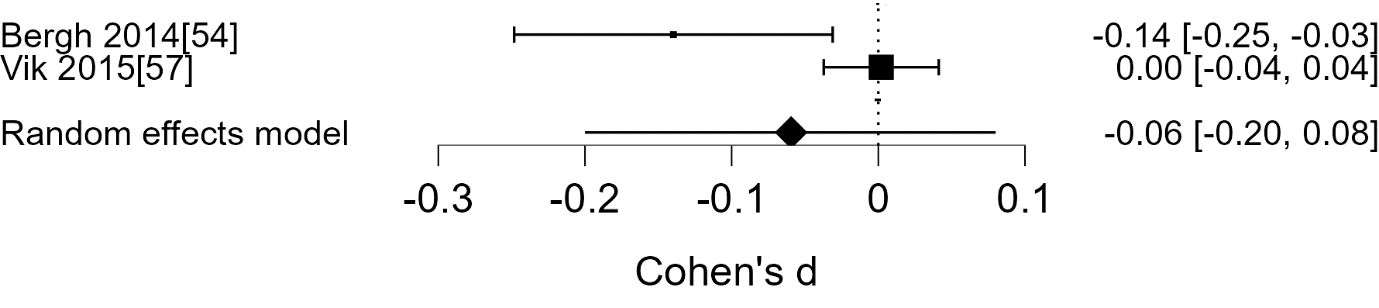


2f.


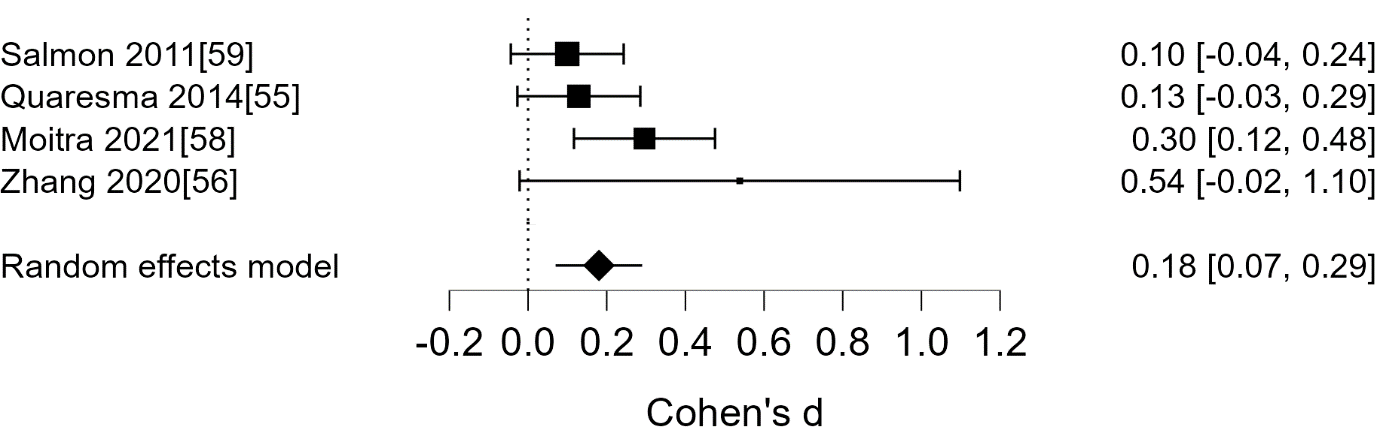


2g.


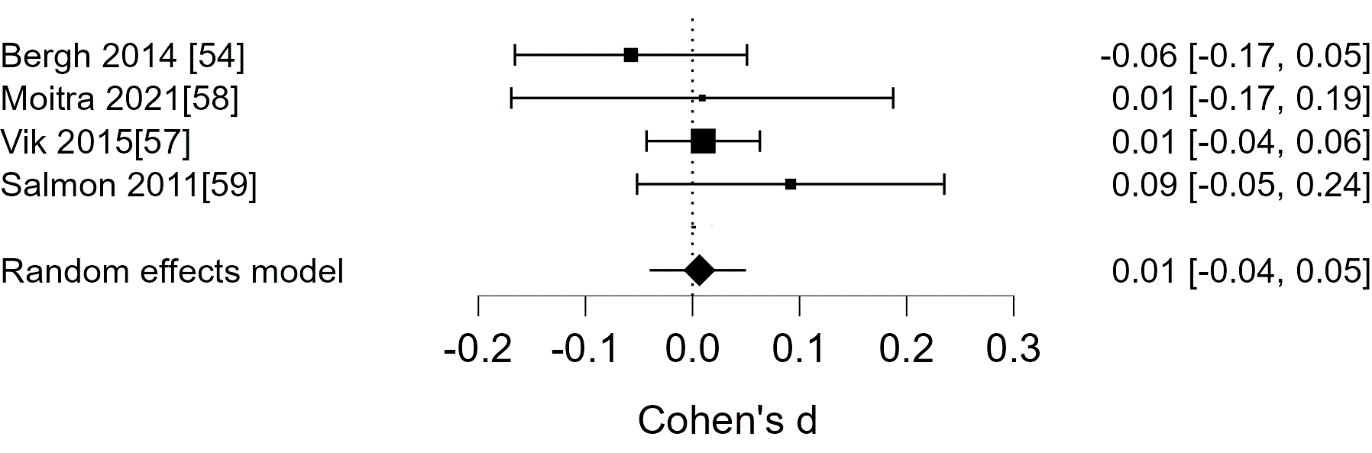


Figure 2. Within school with family/home setting, forest plots showing post-intervention effects on determinants and PAB/SB in RCTs.

(a) Represents self-efficacy in studies targeting PAB and/or SB, *d*=0.25, 95%CI (-0.23, 0.74), *I^2^* = 97.47% (high heterogeneity)

(b) Represents attitude in studies targeting PAB and/or SB, *d*=-0.05, 95%CI (-0.00, 0.10), *I^2^* = 0.00% (low heterogeneity)

(c) Represents subjective norm in studies targeting PAB and/or SB, *d*=-0.12, 95%CI (-0.26, 0.49), *I^2^* = 54.76% (moderate heterogeneity)

(d) Represents knowledge in studies targeting SB, *d*=-0.25, 95%CI (-0.22, 0.72), *I^2^* = 95.69% (high heterogeneity)

(e) Represents parental practice in SB regulation in studies targeting SB, *d*=-0.06, 95%CI (-0.20, 0.08), *I^2^* = 82.72% (high heterogeneity)

(f) Represents overall effect on PAB, *d*=0.18, 95%CI (0.07, 0.29), *I^2^* = 30.04% (low heterogeneity)

(g) Represents overall effect on SB, *d*=0.01, 95%CI (-0.04, 0.05), *I^2^* = 0.00% (low heterogeneity)

1. Cochran’s Q was not used to assess heterogeneity as all MAs are under-powered due to the low number of studies in all MAs (Gavaghan et al., 2000).

   [Gavaghan DJ, Moore, AR, McQay HJ. An evaluation of homogeneity tests in meta-analysis in pain using simulations of patient data. *Pain* 2000; 85: 415-24](http://www.painjournalonline.com/article/S0304-3959(99)00302-4). [↑](#footnote-ref-1)
2. Sterne JAC, Sutton AJ, Ioannidis JPA, et al. Recommendations for examining and interpreting funnel plot asymmetry in meta-analyses of randomised controlled trials. *BMJ* 2011; 343: d4002. [↑](#footnote-ref-2)
