# Supplementary material for: DE-PASS Best Evidence Statement (BESt): Determinants of self-report physical activity and sedentary behaviours in children in settings: A systematic review and meta-analyses
Source: PLoS One. 2024 Nov 25;19(11):e0309890. doi: 10.1371/journal.pone.0309890 (PMC11588252; doi:10.1371/journal.pone.0309890)
Supplement: S1 Appendix — (DOCX) [file pone.0309890.s006.docx]

|  | ES | CI 95%  Lower | CI95%  Upper |
| --- | --- | --- | --- |
| Setting - School only |  |  |  |
| Boyle-Holmes 2010 [47] |  |  |  |
| Motor skill specific self-efficacy (4^th^ grade) (post) | 0.14 | -0.01 | 0.29 |
| Motor skill specific self-efficacy (5^th^ grade) (post) | 0.02 | -0.15 | 0.19 |
| Perception of PA competence (4^th^ grade) (post) | 0.03 | -0.08 | 0.14 |
| Perception of PA competence (5^th^ grade) (post) | -0.04 | -0.15 | 0.07 |
| Habitual PA (4^th^ grade) (post)* | 0.14 | -0.01 | 0.29 |
| Habitual PA (5^th^ grade) (post) | -0.06 | -0.21 | 0.09 |
| Wang 2017 [51] |  |  |  |
| PA self-efficacy (post)* | 0.41 | 0.09 | 0.71 |
| PA self-efficacy (follow-up) | 0.28 | -0.03 | 0.59 |
| PA motivation – autonomous (post) | 0.14 | -0.17 | 0.45 |
| PA motivation – autonomous (follow-up) | 0.12 | -0.19 | 0.43 |
| PA motivation – controlled (post) | 0.04 | -0.27 | 0.34 |
| PA motivation – controlled (follow-up) | -0.02 | -0.33 | 0.59 |
| PA preferences (post) | 0.28 | -0.03 | 0.58 |
| PA preferences (follow-up) | -0.13 | -0.18 | 0.44 |
| Habitual PA (post)* | 0.37 | 0.06 | 0.69 |
| Habitual PA (follow-up)* | 0.40 | 0.09 | 0.72 |
| Gråsten, 2019 [48] |  |  |  |
| PE enjoyment (post) | -0.14 | -0.30 | 0.01 |
| PE enjoyment (follow-up) | 0.00 | -0.16 | 0.16 |
| Habitual PA (post) | 0.17 | -0.05 | 0.39 |
| Habitual PA (follow-up) | 0.14 | -0.08 | 0.36 |
| Gabriel, 2011 [49] |  |  |  |
| Commitment to PA (post) | 0.11 | -0.06 | 0.29 |
| Commitment to PA (follow-up)** | 0.68 | 0.50 | 0.87 |
| Habitual PA (post) | -0.03 | -0.21 | 0.15 |
| Habitual PA (follow-up) | 0.11 | -0.07 | 0.29 |
| Londsdale, 2019 [50] |  |  |  |
| Motivation towards leisure PA – amotivation (post) | -0.06 | -0.17 | 0.05 |
| Motivation towards leisure PA – autonomous (post) | 0.03 | -0.08 | 0.15 |
| Motivation towards leisure PA – controlled (post) | 0.17 | 0.06 | 0.28 |
| Motivation towards PE – amotivation (post) | -0.05 | -0.16 | 0.06 |
| Motivation towards PE – autonomous (post) | 0.04 | -0.07 | 0.15 |
| Motivation towards PE – controlled (post) | -0.00 | -0.11 | 0.11 |
| Needs satisfaction in PE – amotivation (post) | 0.06 | -0.05 | 0.17 |
| Needs satisfaction in PE – autonomous (post) | -0.03 | -0.14 | 0.08 |
| Needs satisfaction in PE – controlled (post) | -0.01 | -0.12 | 0.10 |
| Student perceptions of PE teacher behaviour – controlling (post) | 0.19 | 0.08 | 0.30 |
| Student perceptions of PE teacher behaviour – supporting (post) | 0.01 | -0.10 | 0.11 |
| Motivation towards leisure PA – amotivation (follow-up) | -0.08 | -0.21 | 0.04 |
| Motivation towards leisure PA – autonomous (follow-up) | 0.07 | -0.06 | 0.19 |
| Motivation towards leisure PA – controlled (follow-up) | 0.12 | -0.01 | 0.24 |
| Motivation towards PE – amotivation (follow-up) | 0.01 | -0.11 | 0.13 |
| Motivation towards PE – autonomous (follow-up) | 0.02 | -0.10 | 0.15 |
| Motivation towards PE – controlled (follow-up) | 0.08 | -0.05 | 0.20 |
| Needs satisfaction in PE – amotivation (follow-up) | 0.16 | 0.04 | 0.29 |
| Needs satisfaction in PE – autonomous (follow-up) | 0.01 | -0.12 | 0.13 |
| Needs satisfaction in PE – controlled (follow-up) | 0.02 | -0.10 | 0.15 |
| Student perceptions of PE teacher behaviour – controlling (follow-up)* | 0.25 | 0.12 | 0.37 |
| Student perceptions of PE teacher behaviour – supporting (follow-up) | -0.17 | -0.29 | -0.05 |
| PA duration (post) | 0.08 | -0.04 | 0.21 |
| PA duration (follow-up) | -0.08 | -0.28 | 0.12 |
|  |  |  |  |
| Setting - Family only |  |  |  |
| Maddison 2014 [52] |  |  |  |
| PA enjoyment | -0.04 | -0.30 | 0.23 |
| SB enjoyment | 0.10 | -0.17 | 0.37 |
| Caregiver’s PA | 0.03 | -0.23 | 0.29 |
| PA level | 0.08 | -0.19 | 0.35 |
| Total sedentary time | -0.17 | -0.44 | 0.10 |
|  |  |  |  |
| Setting – School with family/home |  |  |  |
| Pearce 2019 [53] |  |  |  |
| Self-management (post) | 0.12 | -0.25 | 0.49 |
| Perceived barriers to PA (post)* | 0.43 | 0.05 | 0.80 |
| Outcome expectancy (post)* | -0.45 | -0.83 | -0.08 |
| Enjoyment of PA (post) | 0.13 | -0.24 | 0.50 |
| Self-efficacy (post) | -0.11 | -0.48 | 0.26 |
| Social support – home (post) | 0.09 | -0.28 | 0.46 |
| Social support – school (post) | 0.27 | -0.10 | 0.64 |
| Habitual PA (post)* | -0.51 | -0.88 | -0.13 |
| Self-management (follow-up)* | 0.43 | 0.06 | 0.81 |
| Perceived barriers to PA (follow-up) | 0.26 | -0.11 | 0.64 |
| Outcome expectancy (follow-up) | -0.25 | -0.62 | 0.12 |
| Enjoyment of PA (follow-up) | 0.22 | -0.15 | 0.59 |
| Self-efficacy (follow-up) | 0.00 | -0.37 | 0.37 |
| Social support – home (follow-up) | 0.18 | -0.18 | 0.55 |
| Social support – school (follow-up)** | 0.53 | 0.16 | 0.91 |
| Habitual PA (follow-up) | 0.00 | -0.41 | 0.41 |
| Quaresma, 2014 [55] |  |  |  |
| Peer support (post) | 0.15 | -0.01 | 0.31 |
| Teacher support (post) | -0.12 | -0.04 | 0.28 |
| Parental social support (post)* | 0.24 | 0.09 | 0.40 |
| Parental encouragement (post) | 0.08 | -0.08 | 0.23 |
| Amotivation (post) | 0.05 | -0.10 | 0.21 |
| External motivation (post)* | -0.23 | -0.38 | -0.07 |
| Introjected motivation (post) | -0.03 | -0.19 | 0.12 |
| Identified motivation (post) | 0.18 | 0.03 | 0.34 |
| Intrinsic motivation (post) | 0.19 | 0.03 | 0.34 |
| Habitual PA (post) | 0.13 | -0.03 | 0.29 |
| Zhang 2020 [56] |  |  |  |
| Self-efficacy (post)** | 0.71 | 0.13 | 1.35 |
| Outcome expectancy (post) | 0.52 | -0.04 | 1.08 |
| Exercise Attitude (post) | 0.17 | -0.40 | 0.76 |
| Subjective norm (post) | 0.00 | -0.05 | 0.05 |
| Perceived behavioural control (post) | 0.44 | -0.12 | 1.00 |
| Exercise intention (post)*** | 0.87 | 0.30 | 1.45 |
| Habitual PA (post) | 0.54 | -0.03 | 1.15 |
| Bergh 2014 [54] |  |  |  |
| Perceived parental regulation on SB behaviour (post) | -0.14 | -0.25 | -0.03 |
| Screen behaviour | -0.06 | -0.17 | 0.05 |
| Vik 2016 [57] |  |  |  |
| Self-efficacy-SB (post) | -0.04 | -0.11 | 0.03 |
| Attitude-SB (post) | 0.05 | -0.01 | 0.10 |
| Preferences/liking-SB (post) | 0.00 | -0.07 | 0.07 |
| Automaticity-SB (post) | -0.02 | -0.09 | 0.05 |
| Awareness-SB (post) | 0.13 | 0.06 | 0.20 |
| Knowledge-SB (post) | 0.02 | -0.04 | 0.07 |
| Parental practices-SB (post) | 0.00 | -0.04 | 0.04 |
| Parental modeling-SB (post)* | 0.25 | 0.18 | 0.32 |
| Parental subjective norm-SB (post) | 0.00 | -0.05 | 0.05 |
| Availability of TV/DV/PC consoles (post) | 0.3 | -0.04 | 0.10 |
| Sedentary time (post) | 0.01 | -0.04 | 0.06 |
| Salmon 2010 [59] |  |  |  |
| Self-efficacy (post) | 0.11 | -0.01 | 0.23 |
| TV viewing style (post) | 0.44 | -0.12 | 1.00 |
| Sedentary time (post) | 0.09 | -0.05 | 0.24 |
| Moitra 2021 [58] |  |  |  |
| PAB/SB-related knowledge (post)** | 0.50 | 0.31 | 0.68 |
| Habitual PA (post)* | 0.30 | 0.12 | 0.48 |
|  |  |  |  |
| Community with/without other settings |  |  |  |
| Brascum 2013[60] |  |  |  |
| MVPA/SB self-efficacy (post) | 0.33 | -0.02 | 0.68 |
| MVPA/SB self-efficacy (follow-up) | 0.18 | -0.17 | 0.53 |
| MVPA/SB expectations (post) | 0.20 | -0.15 | 0.55 |
| MVPA/SB expectations (follow-up)* | 0.39 | 0.04 | 0.75 |
| MVPA/SB self-control (post) | 0.21 | -0.14 | 0.56 |
| MVPA/SB self-control (follow-up) | 0.18 | -0.17 | 0.53 |
| Habitual PA (post) | 0.24 | -0.23 | 0.71 |
| Habitual PA (follow-up) | 0.02 | -0.45 | 0.48 |
| Sedentary time (post) | 0.24 | -0.17 | 0.77 |
| Sedentary time (follow-up) | 0.34 | -0.13 | 0.80 |
| Christiansen 2014[61] |  |  |  |
| Parents encouragement (post) | 0.09 | -0.03 | 0.22 |
| Perceived safe route to school (post) | -0.14 | -0.27 | -0.02 |
| Attitude to cycling (post) | 0.14 | 0.01 | 0.26 |
| Transportation PA (% active trips to school) (post) | 0.00 | -0.12 | 0.12 |

*Note:* post = post intervention; * denotes small intervention effect (*d*≥ 0.2 <0.5),** denotes moderate effect (*d*≥ 0.5 < 0.8) and ***strong effect (*d*≥ 0.8)*** based on Cohen’s *d*.
